# Perylene-Based Coordination Polymers: Synthesis, Fluorescent J-Aggregates, and Electrochemical Properties

**DOI:** 10.1021/acs.inorgchem.3c00540

**Published:** 2023-05-08

**Authors:** Gonçalo Valente, María Esteve-Rochina, Sergio P. C. Alves, José M. G. Martinho, Enrique Ortí, Joaquín Calbo, Filipe A. Almeida Paz, João Rocha, Manuel Souto

**Affiliations:** † Department of Chemistry, CICECO-Aveiro Institute of Materials, University of Aveiro, Aveiro 3810-393, Portugal; ‡ Instituto de Ciencia Molecular (ICMol), Universidad de Valencia, c/Catedrático José Beltrán, 2, 46980 Paterna, Spain; § Centro de Química Estrutural, Institute of Molecular Sciences and Department of Chemical Engineering, Instituto Superior Técnico, University of Lisbon, 1049-001 Lisbon, Portugal

## Abstract

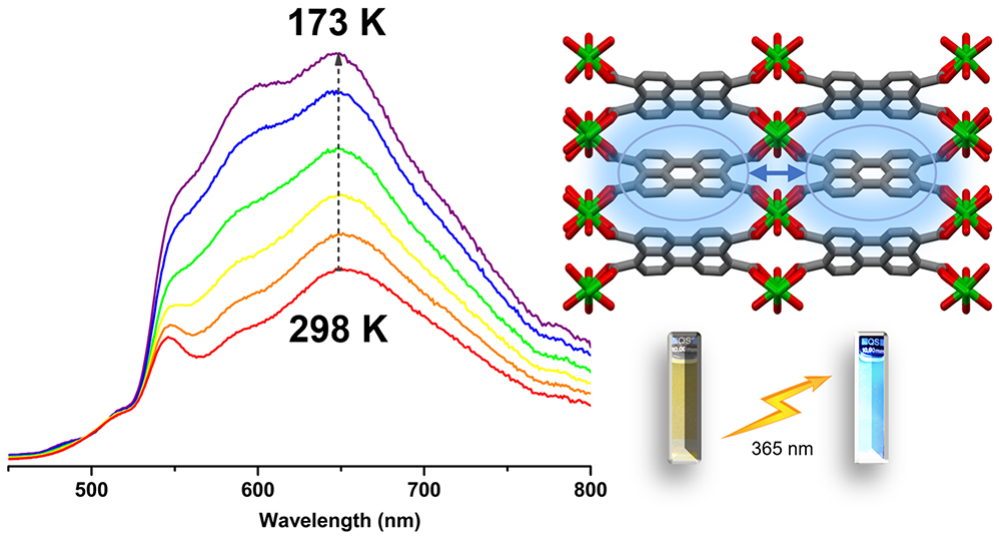

The incorporation of electroactive organic building blocks into coordination polymers (CPs) and metal–organic frameworks (MOFs) offers a promising approach for adding electronic functionalities such as redox activity, electrical conductivity, and luminescence to these materials. The incorporation of perylene moieties into CPs is, in particular, of great interest due to its potential to introduce both luminescence and redox properties. Herein, we present an innovative synthesis method for producing a family of highly crystalline and stable coordination polymers based on perylene-3,4,9,10-tetracarboxylate (PTC) and various transition metals (TMs = Co, Ni, and Zn) with an isostructural framework. The crystal structure of the **PTC-TM** CPs, obtained through powder X-ray diffraction and Rietveld refinement, provides valuable insights into the composition and organization of the building blocks within the CP. The perylene moieties are arranged in a herringbone pattern, with short distances between adjacent ligands, which contributes to the dense and highly organized framework of the material. The photophysical properties of **PTC-Zn** were thoroughly studied, revealing the presence of J-aggregation-based and monomer-like emission bands. These bands were experimentally identified, and their behavior was further understood through the use of quantum-chemical calculations. Solid-state cyclic voltammetry experiments on **PTC-TMs** showed that the perylene redox properties are maintained within the CP framework. This study presents a simple and effective approach for synthesizing highly stable and crystalline perylene-based CPs with tunable optical and electrochemical properties in the solid state.

## Abstract 2

We report the synthesis of a family of isostructural coordination polymers based on perylene-3,4,9,10-tetracarboxylate (PTC) and various transition metals (TMs = Co, Ni, and Zn). The crystal structure of **PTC-TM** CPs reveals that perylene moieties are arranged in a herringbone pattern. The photophysical properties of **PTC-Zn** were thoroughly studied, revealing the presence of J-aggregation-based and monomer-like emission bands. These bands were experimentally identified, and their behavior was further understood through the use of quantum-chemical calculations.

## 1 Introduction

Metal–organic frameworks (MOFs) and coordination polymers (CPs) are hybrid materials constituted by multifunctional organic ligands and metallic nodes, and they present highly ordered and tunable structures.^1,2^ The choice of both the ligands and the metallic nodes plays a crucial role in determining the properties of these crystalline molecular materials. Electroactive organic ligands have been extensively utilized in the design of functional MOFs and CPs that display a range of electronic properties, including conductivity, luminescence, and magnetism, with potential applications in fields such as sensing, electronics, and energy storage.^3,4^

The use of transition-metal (TM)-based complexes and coordination polymers in optoelectronic applications has garnered significant attention due to their tunable optical properties.^5−8^ The photophysical and photochemical properties observed for TM-based coordination polymers are mainly derived from excited-state processes associated with intra-ligand and metal-centered transitions, as well as to metal-to-ligand and ligand-to-metal charge transfers. To gain a comprehensive understanding of the underlying mechanisms of such electronic processes, computational modeling plays a crucial role.^6,9^ Perylenes present remarkable fluorescence properties in diluted solutions, but their luminescence is often suppressed in the solid state due to the formation of π-stacked aggregates, a phenomenon referred to as “aggregation-induced quenching”.^10^ In addition, perylene derivatives such as arylenediimides may exhibit tunable electrochemical properties, becoming very attractive toward a wide range of applications.^11,12^ In recent years, perylene-based ligands have been utilized in the synthesis of luminescent CPs and MOFs by leveraging the isolation of the perylene units or promoting the formation of J-aggregates between them.^10,13−16^ Control over J-type aggregation is a critical aspect to consider as it greatly impacts their performance in various applications such as optoelectronics or light harvesting.^17,18^ Very recently, a coordination polymer based on perylene-3,4,9,10-tetracarboxylate (PTC) and Zn metallic nodes has been reported as a promising biosensor for MicroRNAs detection due to its enhanced electrochemiluminescence.^19^ However, the synthesis of this CP relies on the Zn-for-K metal exchange of a previously reported PTC-K_4_ material,^20^ and although a crystalline material was obtained, the synthesis based on transmetalation may decrease the crystallinity and produce phase impurities and structural defects due to the framework adjustment, especially for non-isostructural materials.^21,22^

Herein, we introduce a novel method to synthesize highly stable and crystalline coordination polymers (CPs) based on the PTC organic unit and different transition metals (TMs = Zn^2+^, Ni^2+^, and Co^2+^). The crystal structure of PTC-TM CPs reveals a dense, nonporous structure in which perylene units are arranged in a herringbone pattern. Powder X-ray diffraction (PXRD) and solid-state NMR confirmed the phase purity of the isostructural materials. The photophysical studies on PTC-Zn CP show a J-type aggregation-induced emission due to the proximity of perylene moieties, as evidenced by both experimental observations and theoretical quantum-chemical calculations. The solid-state electrochemical studies performed on PTC-TM CPs demonstrated that the perylene moieties retain their redox properties within the coordination polymer framework.

## 2 Results and Discussion

### 2.1 Synthesis

**Scheme 1:**
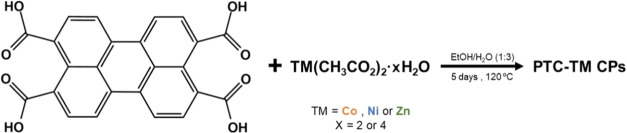
Synthesis of **PTC-TM** CPs

The series of **PTC-TM** (TMs = Co^2+^, Ni^2+^, Zn^2+^) CPs were obtained through a novel synthetic approach based on the direct reaction of the 3,4,9,10-perylenetetracarboxylic acid (H_4_PTCA) and transition-metal (TM) salts (Scheme 1). First, the H_4_PTCA ligand was synthesized from 3,4,9,10-perylenetetracaboxylicdianhydride (PTCDA) (see Scheme S1 and Figures S1 and S2). Then, the **PTC-TM**s were obtained by mixing the ligand H_4_PTCA and the TM(CH_3_COO)_2_·*x*H_2_O (TM = Co^2+^, Ni^2+^, Zn^2+^; *x* = 2 or 4) salts in EtOH/water (1:3) and heating the reaction mixture for 5 days at 120 °C (Scheme 1). The obtained powders were thoroughly washed with dimethylformamide (DMF) and EtOH to remove any unreacted H_4_PTCA ligand, as confirmed by IR spectroscopy (Figure S3). The yields were in the range of 70–80%.

### 2.2 Crystal Structure

**Figure 1:**
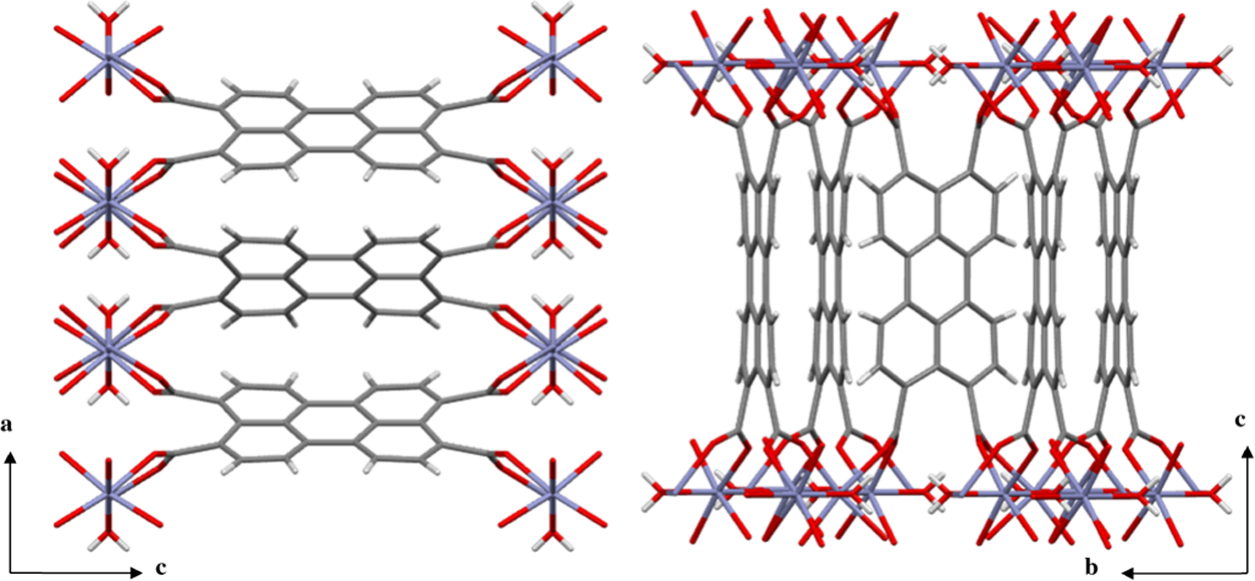
Partial views of the crystal structure of the **PTC-Zn** CP on the *ac* and *bc* planes, showing the arrangement of the Zn^2+^ ions and the PTC linkers. Color code: C (gray), O (red), Zn (blue), H (white).

The crystal structure of the **PTC-TM** CPs was firmly established through powder X-ray diffraction (PXRD). Rietveld refinements were carried out for all materials, and the published structure was used as a comparative model (Figure S4).^19^**PTC-TM**s crystallize in the orthorhombic *Pbam* space group with the 6-coordinated TM^2+^ cations exhibiting a slightly distorted octahedral geometry formed by four carboxylate groups (from four symmetry-related anionic ligands) and two bridging water molecules. Figure 1 shows the crystal packing of **PTC-Zn** as a representative example. The perylene ligands are arranged in a herringbone configuration, similar to that reported in a previous study.^23^ The closest C···C distances between adjacent perylene moieties are estimated to be in the 3.7–3.9 Å range (Figure S5). Distinct from other perylene-based MOFs,^10,14,15,23^ the PTC-TM CPs do not display intrinsic porosity, as evidenced by the lack of solvent-accessible volume in the crystal structure. This characteristic prevents the tuning of their properties through the encapsulation of guest molecules.

### 2.3 PXRD and Solid-State NMR

**Figure 2:**
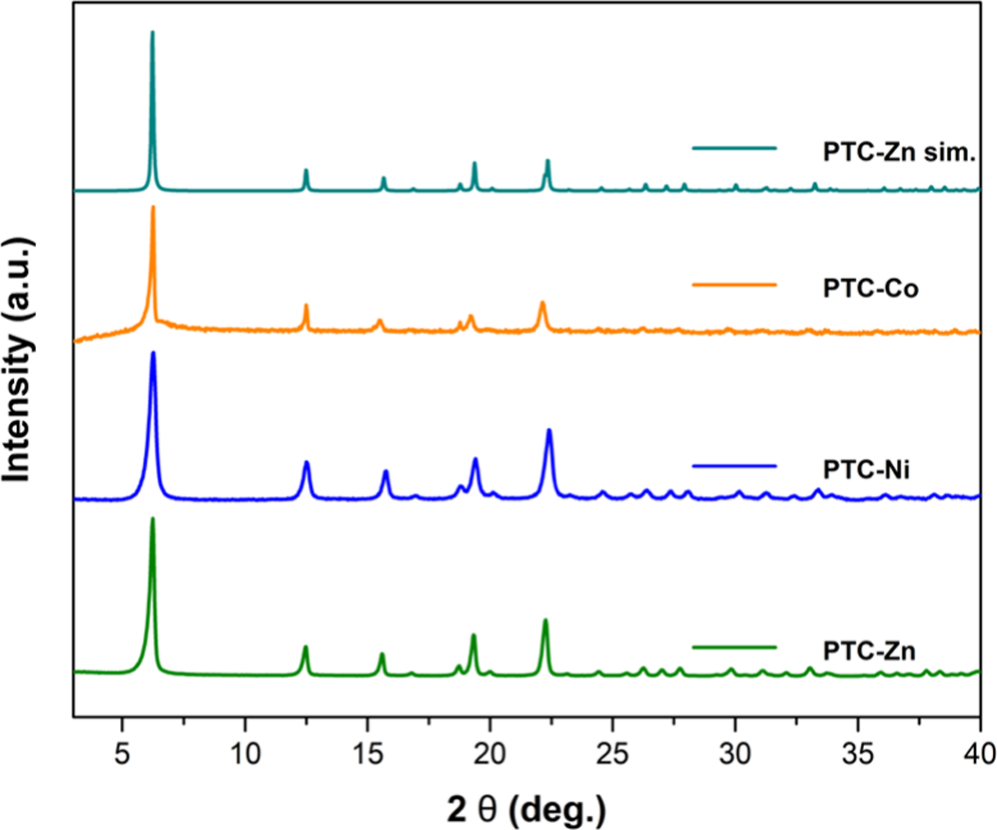
Powder X-ray diffraction patterns of simulated **PTC-Zn** and experimental **PTC-Co**, **PTC-Ni**, and **PTC-Zn** CPs.

The phase purity of the isostructural **PTC-TM** CPs was further confirmed through powder X-ray diffraction (PXRD) studies, where the simulated and experimental patterns were compared (Figure 2). The observation of well-defined peaks with enhanced intensity at 2θ = 6.2° in the PXRD patterns highlights an improvement in the crystallinity of the **PTC-TM** CPs compared to the previously reported **PTC-Zn** material.^19^ The absence of the precursors (H_4_PTCA and TM salts) was also confirmed by PXRD (Figure S6).

**Figure 3:**
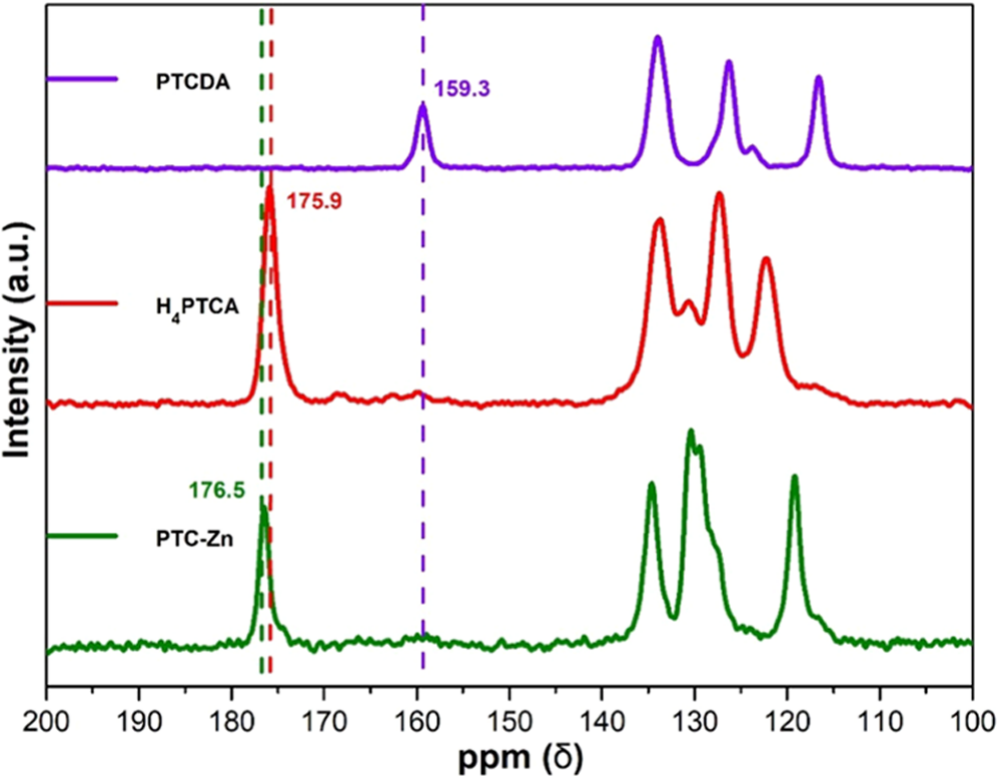
^13^C CPMAS NMR spectra of **PTC-Zn**, H_4_PTCA, and PTCDA.

^13^C solid-state cross-polarization magic-angle spinning (CPMAS) NMR was also performed to show the absence of the PTCDA precursor species and to confirm metal coordination. The ^13^C NMR signals between 115–140 ppm are assigned to the aromatic carbons, whereas the signals at 159.3 and 175.9 ppm are attributed to the anhydride and carboxylic acid groups of PTCDA and H_4_PTCA, respectively (Figure 3). Both the ^13^C CPMAS 175.9 ppm resonance and the absence of a signal at 159.3 ppm support the total conversion of PTCDA to H_4_PTCA, discarding the presence of dianhydride defects. The slight increase in the ^13^C chemical shift of the carboxylate carbon signal upon coordination to Zn^2+^ of H_4_PTCA in the ^13^C CPMAS spectrum of **PTC-Zn** supported metal coordination, although the small difference does not allow to discriminate between different metal coordination modes. In addition, ^1^H MAS NMR spectra also confirmed the coordination of the PTC carboxylic groups to the metal ions (Figure S7).

### 2.4 TGA and SEM

**Figure 4:**
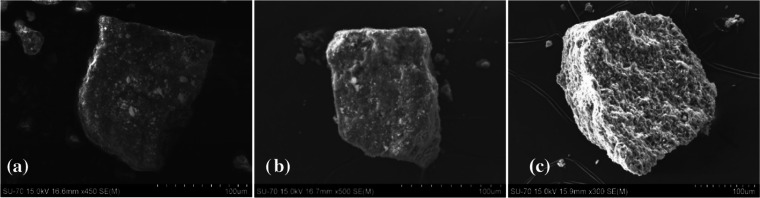
SEM images of (a) **PTC-Co**, (b) **PTC-Ni**, and (c) **PTC-Zn** CP.

The thermal stability of the PTC-TM CPs was analyzed by thermogravimetric analysis (TGA) (Figures S8–S10). The PTC-TM CPs show a 7% mass loss between 100 and 250 °C attributed to the removal of the two water molecules coordinated to the TM^2+^ ions (calculated to be 6% for TM_2_C_24_H_8_O_8_(H_2_O)_2_). The material decomposition begins above 350 °C, showing a relatively high thermal stability. Scanning electron microscopy (SEM) shows PTC-TM CP aggregates with a size of 100–300 μm and a similar morphology (Figures 4 and S11–S13). Energy-dispersive X-ray spectroscopy (EDS) confirms the homogeneous distribution of all elements present in the different materials (Figures S11–S13).

### 2.5 Optical Properties

The optical properties of the **PTC-TM** CPs were characterized by diffuse reflectance UV–vis-NIR spectroscopy in the solid state (Figure S14). The three CPs show similar absorption spectra, with an intense absorption band at 500 nm. The H_4_PTCA ligand displays a strong band at lower energies, as previously reported.^23^ The following optical band gaps (*E*_g_)^24^ were obtained by linearly fitting the absorption onsets in Tauc plots of the Kubelka–Munk–transformed data: 2.33 (**PTC-Co**), 2.34 (**PTC-Ni**), and 2.34 eV (**PTC-Zn**) compared to 2.02 eV for H_4_PTCA (Figure S15). Although the optical band gap values are similar to that recently reported for a perylene-based semiconductor MOF, which exhibits a room-temperature electrical conductivity of σ_RT_ ∼ 10^–8^ S/cm,^23^ in this case, all CPs show lower conductivities (σ_RT_ ∼ 10^–10^ S/cm), measured as pressed pellets.

The emission spectra of **PTC-Co**, **PTC-Ni**, and **PTC-Zn** were measured in the solid state (λ_exc_ = 480 nm). **PTC-Zn** exhibits a significant luminescence (Figure S16) due to the d^10^ closed-shell configuration of Zn(II), whereas the **PTC-Ni** and **PTC-Co** luminescence is almost negligible due to quenching caused by the proximity of the perylene ligand to the paramagnetic ions.^25,26^ Therefore, our photophysical study focuses on **PTC-Zn**.

**Figure 5:**
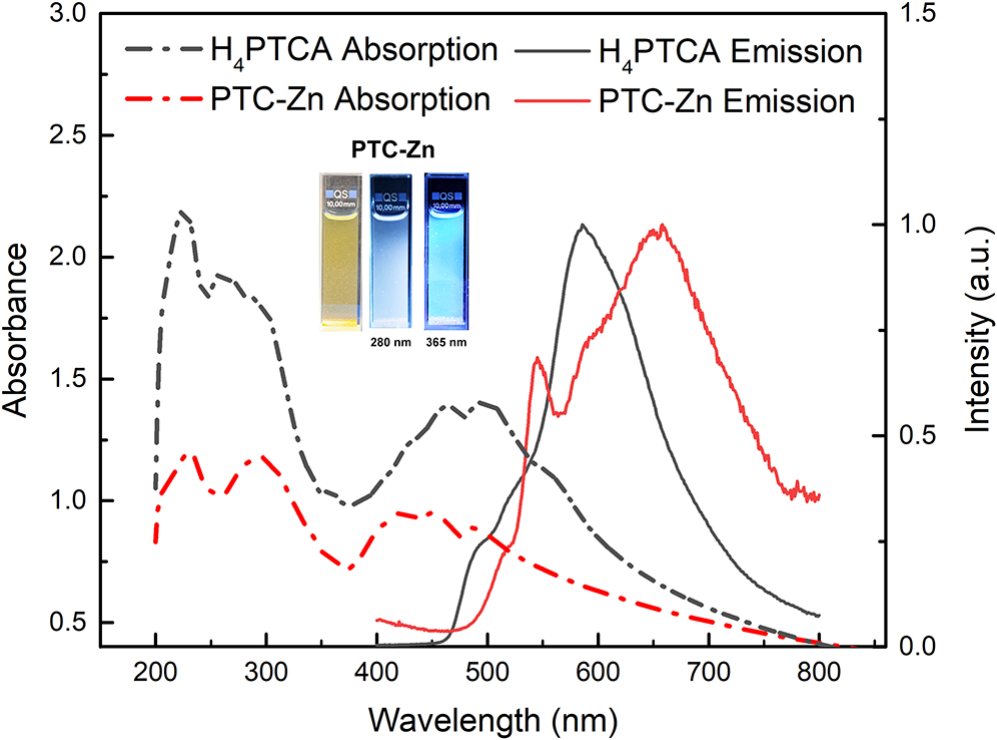
Absorption and emission spectra of H_4_PTCA and **PTC-Zn** suspensions in EtOH (0.08 g L^–1^). The emission spectra were recorded using λ_exc_ = 285 nm. The inset shows the picture of **PTC-Zn** suspensions in ethanol under normal light and upon excitation at λ_exc_ = 280 and 365 nm.

Figure 5 shows the UV–vis absorption and the emission spectra of H_4_PTCA and **PTC-Zn** EtOH suspensions (0.08 g L^–1^). The UV–vis absorption spectra of both compounds exhibit several bands that resemble those of perylene in both the monomer and aggregate state, with a long tail extending into the red region that overlaps the emission. The absorbance is heightened due to light scattering, which increases the optical path of light within the cell. The emission spectra of both compounds consist of two bands: a band with a vibronic structure in the lower wavelength range (500–550 nm) and a broader band at longer wavelengths (600–650 nm). The emission of H_4_PTCA is shifted toward the blue compared to that of **PTC-Zn**, and both spectra are distorted by reabsorption. As the concentration of the dispersion increases, the impact of reabsorption is enhanced (Figure S17), the effect being substantially higher for H_4_PTCA owing to its higher absorption in the emission/absorption overlap region. The optical path of the emitted light also varies with the excitation wavelength, as the distribution of excited molecules within the cell is governed by the Beer–Lambert law, leading to variations in the emission spectrum by changing the excitation wavelength (Figure S17).^27^

**Figure 6:**
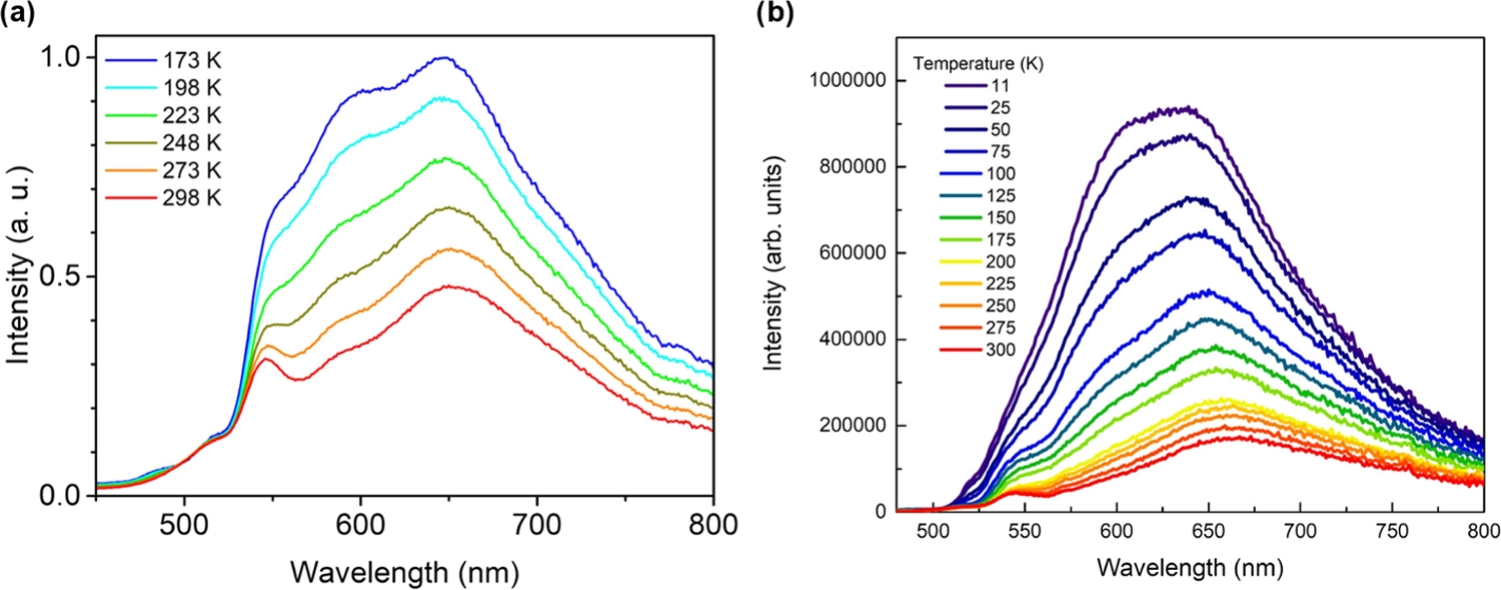
(a) Emission spectra of a **PTC-Zn** suspension in ethanol (λ_exc_ = 285 nm) at different temperatures (173−298 K). Concentration of 0.08 mg mL^–1^. (b) Emission spectra of **PTC-Zn** (λ_exc_ = 285 nm) in the solid state (pressed pellet) at different temperatures (11−300 K).

The UV–vis absorption and the excitation spectra of H_4_PTCA and **PTC-Zn** samples do not match (Figure S18), indicating that a fraction of the excitation light is absorbed by nonemissive species (probably due to H-type aggregation). This may be the reason for the low apparent fluorescence quantum yields observed (lower than 0.3% for both H_4_PTCA and **PTC-Zn** suspensions). Below room temperature, the fluorescence spectra of **PTC-Zn** reveal the presence of two bands. One band, centered at 550 nm, is nearly temperature-insensitive, whereas the other band, at 650 nm, demonstrates increased intensity as the temperature decreases (Figure 6). This suggests that the spectra consist of an almost temperature-invariant emission from perylene monomers in the blue region and a temperature-sensitive and J-aggregation-based emission broad band in the red region.^14^ It is worth noting that the fluorescence of perylene molecules is indeed highly temperature-insensitive due to its high quantum yield, approaching 100% (94% in cyclohexane),^28^ temperature-dependent nonradiative deactivation processes being virtually absent. The emission from perylene derivatives is therefore dominated by the radiative process, which varies slightly with temperature. Conversely, the intensity of the J-aggregation-like band increases by decreasing temperature owing to the slowdown of the disorder induced by temperature, enabling an efficient exciton coupling.^29^

**Figure 7:**
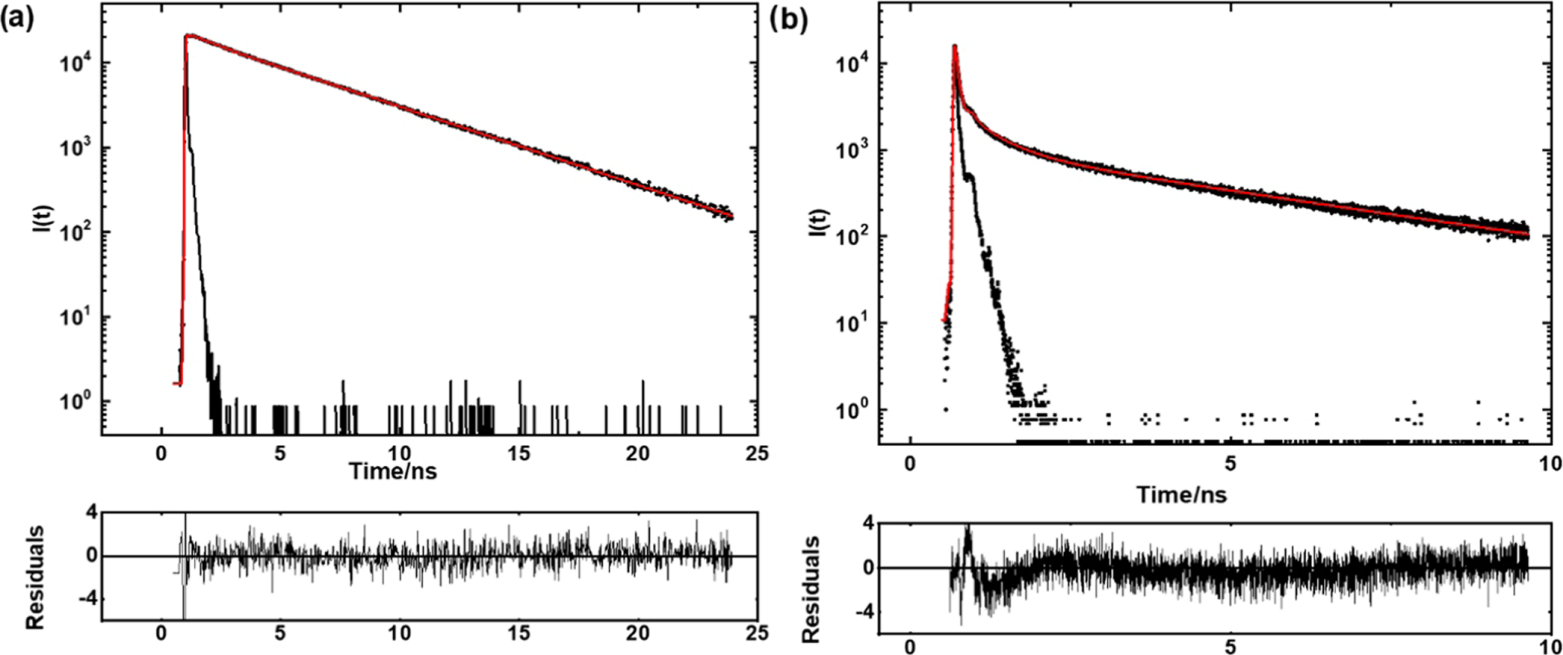
Fluorescence decay curves of (a) H_4_PTCA and (b) **PTC-Zn** suspensions in ethanol (0.08 g L^–1^) by excitation at λ_exc_ = 285 nm and recording the emission at λ_em_ = 590 nm.

This behavior was further confirmed by the fluorescence decay curves shown in Figure 7a (H_4_PTCA) and Figure 7b (**PTC-Zn**) measured in ethanol suspensions (0.08 g L^–1^) at room temperature, with 285 nm excitation and recording the emission at 590 nm. The decays can be fitted to a sum of three exponentials (*I*(*t*) = ∑_*i* = 1_^3^*a*_*i*_ × exp (−*t*/τ_*i*_)) with a very short lifetime (<0.1 ns) component attributed to light scattering. The decay components are *a*_1_ = 0.48, τ_1_ = 0.03 ns; *a*_2_ = 0.05, τ_2_ = 0.94 ns; and *a*_3_ = 0.47, τ_3_ = 4.7 ns with χ^2^ = 1.5 for H_4_PTCA; and *a*_1_ = 0.88, τ_1_ = 0.03 ns; *a*_2_ = 0.08, τ_2_ = 0.50 ns; and *a*_3_ = 0.04, τ_3_ = 3.8 ns with χ^2^ = 1.4 for **PTC-Zn**. The shortest component (0.94 ns for H_4_PTCA and 0.50 ns for **PTC-Zn**) is assigned to the perylene excimer-like emission of the J-aggregates^14^ while the longest component (4.7 ns for H_4_PTCA and 3.8 ns for **PTC-Zn**) is ascribed to the perylene monomer.^17^ The excimer-like emission is attributed to J-aggregates based on its short lifetime (∼1 ns) compared to the dimer excimer emission with 17.6 ns in toluene and 19 ns in the solid state.^30,31^

Irrespective of the excitation and emission wavelengths, the decay curves can always be fitted with a sum of two exponentials with similar lifetimes, indicating the presence of only two species (perylene monomer and J-aggregates perylene excimer-like emission plus a scattering component). The decay pre-exponential factors vary with the emission wavelength, reflecting the contribution of the perylene monomer and perylene J-aggregation-based bands to global emission. The fact that the lifetimes are similar for all emission wavelengths (the differences can be explained by the effect of reabsorption) with no appearance of a rise-time component for longer wavelengths indicates that reabsorption primarily occurs through nonemissive species.^32^

### 2.6 Quantum-Chemical Calculations

**Figure 8:**
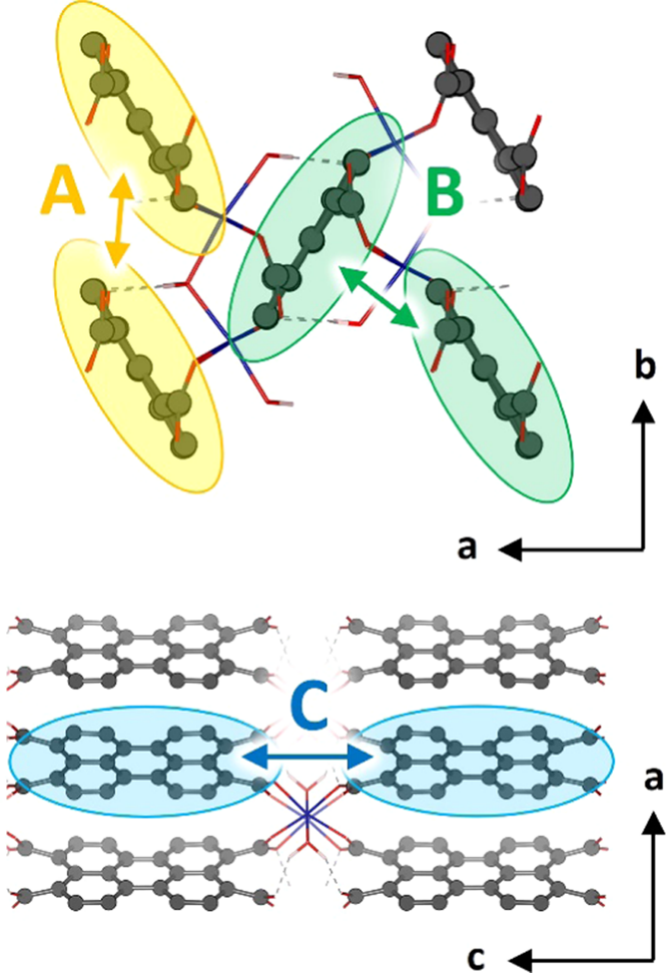
Schematic representation of the PTC···PTC types of interactions in **PTC-TM** CPs: parallel-displaced (A), T-shaped (B), and long-ranged lateral (C).

The minimum-energy crystal structure of **PTC-Co**, **PTC-Ni**, and **PTC-Zn** was obtained upon full ionic and lattice relaxation within the density functional theory (DFT) approach, using the PBEsol functional^33^ and the light tier-1 basis set, and starting from the experimentally resolved X-ray data. Dispersion corrections were included by means of the Tkatchenko and Scheffler formulation.^34^ Spin moment was set to the most stable configuration (Table S1): diamagnetic for **PTC-Zn** and ferromagnetic ordering for **PTC-Ni** and **PTC-Co** (Co^2+^ in high spin) (see the Supporting Information for full computational details). As previously noted from the experimental X-ray data, theoretical minimum-energy structures show that the metal coordination is slightly distorted from octahedral, particularly for **PTC-Co** (Table S2). The predicted crystal structure parameters have a strong correlation with the experimental data, especially for lattice angles and the longest *c*-length. However, a systematic deviation is obtained for the *a*- and *b*-lengths, which are calculated to be 0.2–0.3 Å shorter with respect to the X-ray structure (Table S3). Thermal expansion, which is not considered due to computational expense, is expected to increase the *a* and *b* lattice parameters and be the main factor for such a shift. Each PTC ligand interacts with the neighboring PTCs in three different ways along the **PTC-TM** crystal (Figure 8): parallel-displaced type-A stack, with a perylene centroid···centroid distance (*d*_cc_) of *ca.* 6.7 Å and closest π–π interaction at 3.6 Å; T-shaped type-B stack, with *d*_cc_ = 5.8 Å and closest C-H···π distance of 2.7 Å; and long-ranged lateral type-C stack, with *d*_cc_ = 14.2 Å (Table S4). The following discussion will focus on the three types of stacking interactions and their impact on the optical properties of **PTC-TM**.

**Figure 9:**
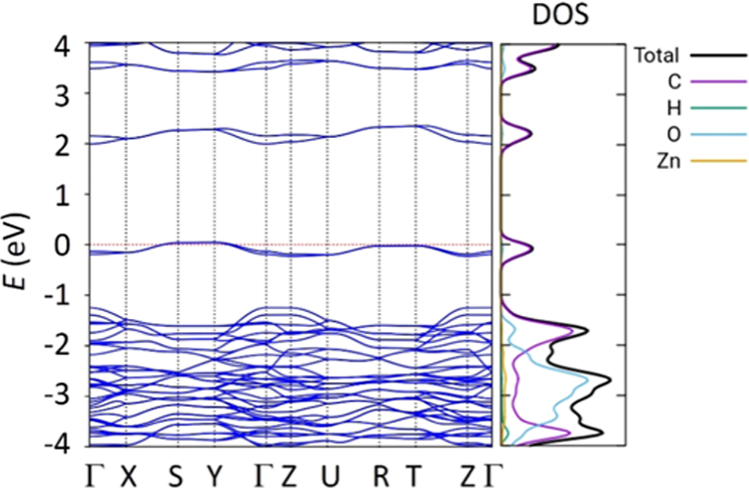
Band structure and atom-projected density of states (DOS) calculated for **PTC-Zn** at the HSE06/light tier-1 level of theory. The Fermi level was set to the top of the valence band.

Band structure and density of states (DOS) DFT calculations, using the HSE06 functional and the light tier-1 basis set, were performed on the fully relaxed crystal structures to unveil the electronic, optical, and redox properties of the perylene-based CP materials. A full *k*-path in the *Pbam* first Brillouin zone along Γ–X–S–Y−Γ–Z–U–R–T–Z−Γ and a 3 × 3 × 3 *k*-grid were employed. Theoretical calculations predict a band structure with moderate dispersion for the valence and conduction bands, especially in the *k*-segments located within the *ab*-plane (Figure 9 for **PTC-Zn** and Figure S19 for **PTC-Co** and **PTC-Ni**). This region is where perylene units stack to form T-shaped and parallel-displaced arrangements (Figure 8). Atom-projected DOS indicates that the valence band maximum (VBM) and conduction band minimum (CBM) energy levels originate from the perylene moiety (Figure 9). The topology of the highest-occupied crystal orbital (HOCO) and the lowest-unoccupied crystal orbital (LUCO) (as seen in Figure S20) actually corresponds to that calculated for the highest-occupied molecular orbital (HOMO) and lowest-unoccupied molecular orbital (LUMO) of pristine H_4_PTCA (as seen in Figure S21). Consequently, the predicted band gap is very similar in all three CPs, with little effect from the type of metal atom: 2.22(α)/2.16(β) eV for **PTC-Co**, 2.17(α)/2.11(β) eV for **PTC-Ni**, and 2.13 eV for **PTC-Zn**, in line with the experimental data (Figure S15). In these materials, the oxidation and reduction processes are thus expected to occur at the perylene moiety. Unlike **PTC-Ni** and **PTC-Zn**, **PTC-Co** displays energy levels close to the VBM originating from the metal atom, which are calculated at 0.5–1.0 eV below the Fermi level (see the HOCO-2(β) topology corresponding to a *d*-orbital of Co in Figure S22). This suggests that the oxidation of Co(II) to Co(III) is possible at positive potential values near the oxidation potential of PTC (as described below in Section 2.7).

To qualitatively evaluate the potential of these materials in next-generation applications, electrical conductivity was assessed by calculating the effective masses along the full *k*-path, obtaining values as small as 0.96 m_0_ and 0.93 m_0_ for hole and electron, respectively, in **PTC-Zn** (*k*-segment: U–R; Table S6). Although the experimental results for conductivity are not encouraging, theoretical calculations suggest that there is moderate charge transport along the perylene stacks, particularly along the *b*-axis (which contains a parallel-displaced arrangement between PTCs with a π–π intermolecular distance of approximately 3.6 Å, A interaction in Figure 8) after carriers are generated. However, it is important to note that the lack of permanent porosity in these CPs prevents the generation of charge carriers through guest engineering,^21^ thereby limiting their potential use as conducting materials.

To shed light on the absorption and emission properties of our CPs, theoretical calculations were performed in a multilevel approach considering molecular systems, dimeric models, and periodic crystal structures. The H_4_PTCA ligand presents a HOMO–LUMO gap of 2.37 eV, in good accord with the experimental band gap values for **PTC-TM** materials (2.33–2.34 eV). The vibrationally resolved absorption spectrum of H_4_PTCA calculated using the time-dependent (TD) DFT approach displays the typical vibrational progression with the most intense 0–0 transition at 520 nm (2.38 eV; Figure S25). This information is in good agreement with the experimental absorption peak recorded at around 500 nm (2.48 eV) and the vibrational structure of the excitation spectrum of H_4_PTCA and **PTC-Zn** (Figure S18).

The excitonic coupling between the three types of PTC···PTC interactions was estimated (Table S4) to unveil their effect on the optical properties of **PTC-TM** CPs. Despite being small, a negative excitonic coupling of about −30 meV, indicative of J-aggregation, is calculated for the C-type dimer of **PTC-TM**s (Figure 8). This may explain the experimental absorption shoulder found at 550 nm (2.25 eV) for H_4_PTCA in the solid state, and also present (but much less intense) for **PTC-TM** (Figures S14 and S15).^18^ On the other hand, type-A and type-B dimers are calculated with excitonic couplings of +60 and +70 meV, respectively, accounting for the presence of H-type aggregation, in line with the low-lying broad absorption signal (*ca.* 500 nm, Figure 5; with 0–0/0–1 vibronic intensity inversion in the case of **PTC-Zn**), and supporting the possibility of the formation of long-range excimer species. Overall, the predicted absorption spectrum calculated for the crystal structure of **PTC-TM**s via the linear macroscopic dielectric function approximation at the HSE06/light tier-1 level nicely matches the experiments (*cf*. Figures 5 and S26).

To confirm the aggregation-induced excimer/exciplex nature of the broad emission band recorded experimentally at 600–700 nm in **PTC-Zn**, TD-DFT calculations were performed for type-A, type-B, and type-C PTC dimers as extracted from the crystalline structure of **PTC-Zn** (see the SI for computational details). Theoretical calculations at the PBE0/6-31G(d,p) level indicate that the lowest-lying singlet excited state is mainly described by a charge-transfer (CT) excitation from one PTC to the other (Figure S25). Because the perylene···perylene centroid distance is expected to remain constant during excimer formation due to the rigidity of the framework and the innocent nature of the inorganic node during the transition, CT excited-state relaxation was simulated in the dimer by replacing the structure of the neutral PTC monomers by the optimized geometry of a positively charged and a negatively charged PTC. Theoretical calculations predict that the lowest-lying S_1_ state lies 2.22, 1.84, and 1.87 eV (559, 673, and 663 nm, respectively) above the ground state for dimers A, B, and C, respectively. These values are in good agreement with the J-aggregation-based broad emission band observed experimentally at ca. 675 nm for **PTC-Zn** with a shoulder at ca. 600 nm (Figure 5).

### 2.7 Cyclic Voltammetry

**Figure 10:**
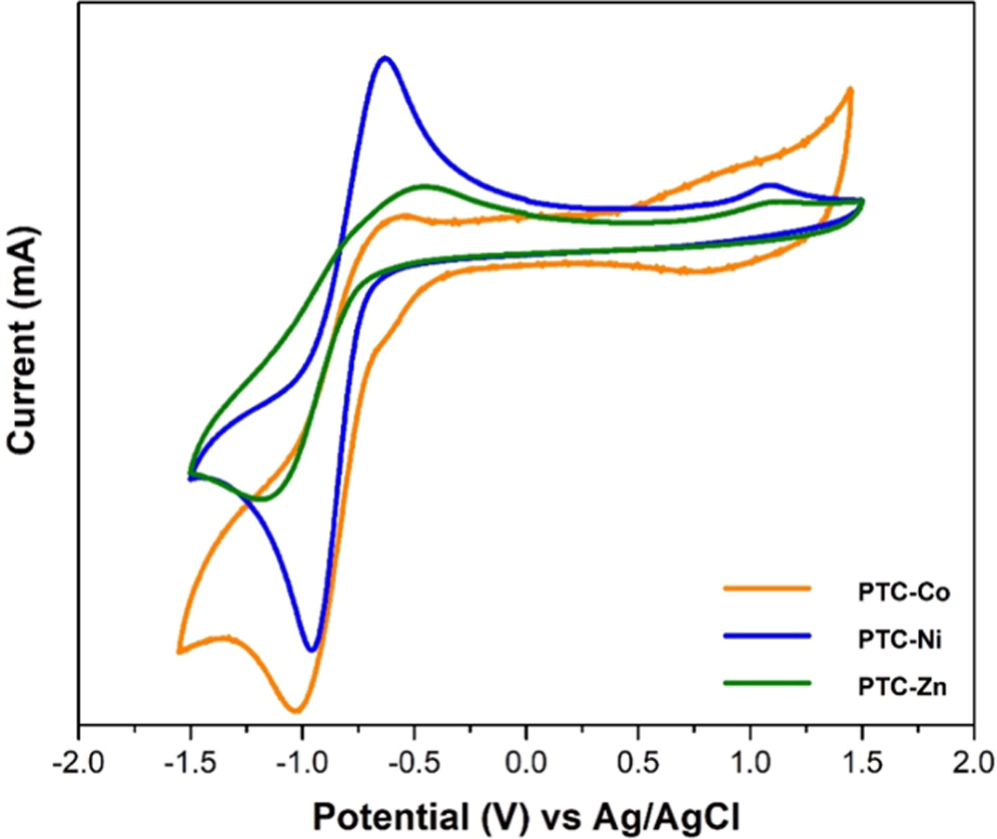
Solid-state cyclic voltammetry of **PTC-Co**, **PTC-Ni**, and **PTC-Zn** CPs in CH_3_CN using TBAPF_6_ 0.1 M as the electrolyte at 0.5 V s^–1^ scan rate. A platinum wire was used as the counter electrode, and a silver wire as the pseudo-reference electrode. Ferrocene was added as an internal standard. All potentials are quoted versus Ag/AgCl.

Solid-state cyclic voltammetry (CV) of **PTC-TM** CPs was performed at room temperature in CH_3_CN using TBAPF_6_ 0.1 M as an electrolyte at different scan rates to evaluate the redox activity of the perylene-based materials (Figure 10). The CV of the H_4_PTCA ligand measured as a reference in DMF (Figure S26) exhibits a reversible redox process at −0.85 V (vs Ag/AgCl) assigned to the reduction of H_4_PTCA,^35^ and a more irreversible redox process at +1.10 V attributed to the oxidation of the perylene moieties to the radical cation state.^36^**PTC-Co**, **PTC-Ni**, and **PTC-Zn** show similar reduction processes at −0.80, −0.79, and −0.82 V, respectively. The peak-to-peak separation between the cathodic and anodic peaks is in the range of Δ*E*_p_ = 300–500 mV, whereas the variation of the cathodic peak current *i*_pc_ (mA) with the scan rate ν (V s^–1^) is almost linear (Figures S27–S29). The peak-to-peak separation slightly increases with the scan rate, suggesting that the reduction process is electrochemically quasi-reversible.^37^ The reversible oxidation peak observed for **PTC-Co** around +0.8 V is due to the Co(II)-to-Co(III) redox process, as evidenced by the theoretical calculations and the observation of similar redox processes in cobalt complexes.^38,39^ This is in contrast to **PTC-Ni** and **PTC-Zn** CPs, which only exhibit an irreversible oxidation peak at +1.08 V (vs Ag/AgCl) due to the perylene ligand oxidation.

## 3 Conclusions

In conclusion, we present a simple and effective method for synthesizing a new family of isostructural transition-metal coordination polymers based on the electroactive perylene building block (**PTC-TM**, TMs = Co, Ni, and Zn). The novel perylene-based CPs exhibit high crystallinity and stability, as evidenced by multiple techniques such as powder X-ray diffraction, solid-state NMR, and thermogravimetric analysis. The photophysical properties of **PTC-Zn** were thoroughly characterized, confirming the coexistence of J-aggregation-based and monomer-like emissions with lifetimes of 0.5 and 3.8 ns, respectively. This observation is nicely supported by quantum-chemical calculations showing that both the absorption and emission properties of **PTC-TM**s are determined by the perylene-based ligand, whose interaction between vicinal PTCs gives rise to the J-type aggregation-induced broad red emission band observed in the 600–700 nm region. Furthermore, solid-state cyclic voltammetry of **PTC-TM** CPs demonstrated that the perylene moieties can undergo reduction and oxidation within the framework, exhibiting an electrochemical behavior similar to that of the free ligand. Our work showcases the versatility of perylene-based coordination polymers and metal–organic frameworks in controlling fluorescence properties by manipulating linker orientation, as well as tuning redox-active properties through the selection of appropriate building blocks. This research therefore opens up new avenues for the development of advanced materials with improved optical and electrochemical properties for a wide range of applications.

## Supporting Information Available

General methods and materials, synthesis and characterization of H_4_PTCA ligand, synthesis of PTC-TM CPs, characterization of PTC-TM CPs (FT-IR, PXRD, TGA, SEM, optical properties, solid-state cyclic voltammetry), and computational details.
Crystal structures (CIF files) of **PTC-Zn**, **PTC-Ni**, and **PTC-Co** (PDF)

## References

[ref1] Maurin G.; Serre C.; Cooper A.; Férey G. The New Age of MOFs and of Their Porous-Related Solids. Chem. Soc. Rev. 2017, 46, 3104–3107. 10.1039/C7CS90049J. 28561090

[ref2] Dincă M.; Long J. R. Introduction: Porous Framework Chemistry. Chem. Rev. 2020, 120, 8037–8038. 10.1021/acs.chemrev.0c00836. 32842743

[ref3] Souto M.; Strutyński K.; Melle-Franco M.; Rocha J. Electroactive Organic Building Blocks for the Chemical Design of Functional Porous Frameworks (MOFs and COFs) in Electronics. Chem. Eur. J. 2020, 26, 10912–10935. 10.1002/chem.202001211. 32293769

[ref4] Ding B.; Solomon M. B.; Leong C. F.; D’Alessandro D. M. Redox-Active Ligands: Recent Advances towards Their Incorporation into Coordination Polymers and Metal-Organic Frameworks. Coord. Chem. Rev. 2021, 439, 213891 10.1016/j.ccr.2021.213891.

[ref5] Choy W. C. H.; Chan W. K.; Yuan Y. Recent Advances in Transition Metal Complexes and Light-Management Engineering in Organic Optoelectronic Devices. Adv. Mater. 2014, 26, 5368–5399. 10.1002/adma.201306133. 25042158

[ref6] Fumanal M.; Corminboeuf C.; Smit B.; Tavernelli I. Optical Absorption Properties of Metal–Organic Frameworks: Solid State versus Molecular Perspective. Phys. Chem. Chem. Phys. 2020, 22, 19512–19521. 10.1039/D0CP03899G. 32839805

[ref7] Parmar B.; Bisht K. K.; Rachuri Y.; Suresh E. Zn(ii)/Cd(ii) Based Mixed Ligand Coordination Polymers as Fluorosensors for Aqueous Phase Detection of Hazardous Pollutants. Inorg. Chem. Front. 2020, 7, 1082–1107. 10.1039/C9QI01549C.

[ref8] Patel U.; Parmar B.; Dadhania A.; Suresh E. Zn(II)/Cd(II)-Based Metal–Organic Frameworks as Bifunctional Materials for Dye Scavenging and Catalysis of Fructose/Glucose to 5-Hydroxymethylfurfural. Inorg. Chem. 2021, 60, 9181–9191. 10.1021/acs.inorgchem.1c01208. 34096303

[ref9] Capano G.; Ambrosio F.; Kampouri S.; Stylianou K. C.; Pasquarello A.; Smit B. On the Electronic and Optical Properties of Metal–Organic Frameworks: Case Study of MIL-125 and MIL-125-NH 2. J. Phys. Chem. C 2020, 124, 4065–4072. 10.1021/acs.jpcc.9b09453.

[ref10] Li J.; Yuan S.; Qin J. S.; Huang L.; Bose R.; Pang J.; Zhang P.; Xiao Z.; Tan K.; Malko A. V.; Cagin T.; Zhou H. C. Fluorescence Enhancement in the Solid State by Isolating Perylene Fluorophores in Metal-Organic Frameworks. ACS Appl. Mater. Interfaces 2020, 12, 26727–26732. 10.1021/acsami.0c05512. 32406228

[ref11] Kumar Y.; Kumar S.; Bansal D.; Mukhopadhyay P. Synthesis and Isolation of a Stable Perylenediimide Radical Anion and Its Exceptionally Electron-Deficient Precursor. Org. Lett. 2019, 21, 2185–2188. 10.1021/acs.orglett.9b00490. 30869910

[ref12] Shukla J.; Singh V. P.; Mukhopadhyay P. Molecular and Supramolecular Multiredox Systems. ChemistryOpen 2020, 9, 304–324. 10.1002/open.201900339. 32154051 PMC7050954

[ref13] Dietl C.; Hintz H.; Rühle B.; Auf Der Ginne S. J.; Langhals H.; Wuttke S. Switch-On Fluorescence of a Perylene-Dye-Functionalized Metal-Organic Framework through Postsynthetic Modification. Chem. - Eur. J. 2015, 21, 10714–10720. 10.1002/chem.201406157. 26037475

[ref14] Sikdar N.; Dutta D.; Haldar R.; Ray T.; Hazra A.; Bhattacharyya A. J.; Maji T. K. Coordination-Driven Fluorescent J-Aggregates in a Perylenetetracarboxylate-Based MOF: Permanent Porosity and Proton Conductivity. J. Phys. Chem. C 2016, 120, 13622–13629. 10.1021/acs.jpcc.6b04347.

[ref15] Seco J. M.; San Sebastián E.; Cepeda J.; Biel B.; Salinas-Castillo A.; Fernández B.; Morales D. P.; Bobinger M.; Gómez-Ruiz S.; Loghin F. C.; Rivadeneyra A.; Rodríguez-Diéguez A. A Potassium Metal-Organic Framework Based on Perylene-3,4,9,10-Tetracarboxylate as Sensing Layer for Humidity Actuators. Sci. Rep. 2018, 8, 14414 10.1038/s41598-018-32810-7. 30258083 PMC6158245

[ref16] Mo F.; Han Q.; Chen M.; Meng H.; Guo J.; Fu Y. Novel Optoelectronic Metal Organic Framework Material Perylene Tetracarboxylate Magnesium: Preparation and Biosensing †. Nanoscale 2021, 13, 16244–16250. 10.1039/d1nr03300j. 34549218

[ref17] Kaiser T. E.; Wang H.; Stepanenko V.; Würthner F. Supramolecular Construction of Fluorescent J-Aggregates Based on Hydrogen-Bonded Perylene Dyes. Angew. Chem., Int. Ed. 2007, 46, 5541–5544. 10.1002/anie.200701139. 17579911

[ref18] Spano F. C. The Spectral Signatures of Frenkel Polarons in H- and J-Aggregates. Acc. Chem. Res. 2010, 43, 429–439. 10.1021/ar900233v. 20014774

[ref19] Wang J.-M.; Yao L.-Y.; Huang W.; Yang Y.; Liang W.-B.; Yuan R.; Xiao D.-R. Overcoming Aggregation-Induced Quenching by Metal–Organic Framework for Electrochemiluminescence (ECL) Enhancement: Zn-PTC as a New ECL Emitter for Ultrasensitive MicroRNAs Detection. ACS Appl. Mater. Interfaces 2021, 13, 44079–44085. 10.1021/acsami.1c13086. 34514796

[ref20] Huang M.; Schilde U.; Kumke M.; Antonletti M.; Cölfen H. Polymer-Induced Self-Assembly of Small Organic Molecules into Ultralong Microbelts with Electronic Conductivity. J. Am. Chem. Soc. 2010, 132, 3700–3707. 10.1021/ja906667x. 20187639

[ref21] Lalonde M.; Bury W.; Karagiaridi O.; Brown Z.; Hupp J. T.; Farha O. K. Transmetalation: Routes to Metal Exchange within Metal–Organic Frameworks. J. Mater. Chem. A 2013, 1, 5453 10.1039/c3ta10784a.

[ref22] Canossa S.; Fornasari L.; Demitri N.; Mattarozzi M.; Choquesillo-Lazarte D.; Pelagatti P.; Bacchi A. MOF Transmetalation beyond Cation Substitution: Defective Distortion of IRMOF-9 in the Spotlight. CrystEngComm 2019, 21, 827–834. 10.1039/C8CE01808A.

[ref23] Valente G.; Esteve-Rochina M.; Paracana A.; Rodríguez-Diéguez A.; Choquesillo-Lazarte D.; Ortí E.; Calbo J.; Ilkaeva M.; Mafra L.; Hernández-Rodríguez M. A.; Rocha J.; Alves H.; Souto M. Through-Space Hopping Transport in an Iodine-Doped Perylene-Based Metal–Organic Framework. Mol. Syst. Des. Eng. 2022, 7, 1065–1072. 10.1039/D2ME00108J.

[ref24] Fabrizio K.; Le K. N.; Andreeva A. B.; Hendon C. H.; Brozek C. K. Determining Optical Band Gaps of MOFs. ACS Mater. Lett. 2022, 4, 457–463. 10.1021/acsmaterialslett.1c00836.

[ref25] Bauer C. A.; Jones S. C.; Kinnibrugh T. L.; Tongwa P.; Farrell R. A.; Vakil A.; Timofeeva T. V.; Khrustalev V. N.; Allendorf M. D. Homo- and Heterometallic Luminescent 2-D Stilbene Metal–Organic Frameworks. Dalton Trans. 2014, 43, 2925–2935. 10.1039/C3DT52939H. 24346232

[ref26] Pamei M.; Puzari A. Luminescent Transition Metal–Organic Frameworks: An Emerging Sensor for Detecting Biologically Essential Metal Ions. Nano-Struct. Nano-Objects 2019, 19, 100364 10.1016/j.nanoso.2019.100364.

[ref27] Martinho J. M. G.; Maçanita A. L.; Berberan-Santos M. N. The Effect of Radiative Transport on Fluorescence Emission. J. Chem. Phys. 1989, 90, 53–59. 10.1063/1.456504.

[ref28] Brandy L. E. Handbook of Fluorescence Spectra of Aromatic Molecules; Academic Press, Inc.: London, 1966; Vol. 9 10.1021/jm00324a069.

[ref29] Kistler K. A.; Pochas C. M.; Yamagata H.; Matsika S.; Spano F. C. Absorption, Circular Dichroism, and Photoluminescence in Perylene Diimide Bichromophores: Polarization-Dependent H- and J-Aggregate Behavior. J. Phys. Chem. B 2012, 116, 77–86. 10.1021/jp208794t. 22171650

[ref30] Katoh R.; Sinha S.; Murata S.; Tachiya M. Origin of the Stabilization Energy of Perylene Excimer as Studied by Fluorescence and Near-IR Transient Absorption Spectroscopy. J. Photochem. Photobiol. A: Chem. 2001, 145, 23–34. 10.1016/S1010-6030(01)00562-7.

[ref31] Ni W.; Sun L.; Gurzadyan G. G. Ultrafast Spectroscopy Reveals Singlet Fission, Ionization and Excimer Formation in Perylene Film. Sci. Rep. 2021, 11, 5220 10.1038/s41598-021-83791-z. 33664304 PMC7933242

[ref32] Nunes Pereira E. J.; Berberan-Santos M. N.; Fedorov A.; Vincent M.; Gallay J.; Martinho J. M. G. Molecular Radiative Transport. III. Experimental Intensity Decays. J. Chem. Phys. 1999, 110, 1600–1610. 10.1063/1.477800.

[ref33] Perdew J. P.; Ruzsinszky A.; Csonka G. I.; Vydrov O. A.; Scuseria G. E.; Constantin L. A.; Zhou X.; Burke K. Restoring the Density-Gradient Expansion for Exchange in Solids and Surfaces. Phys. Rev. Lett. 2008, 100, 136406 10.1103/PhysRevLett.100.136406. 18517979

[ref34] Tkatchenko A.; Scheffler M. Accurate Molecular van Der Waals Interactions from Ground-State Electron Density and Free-Atom Reference Data. Phys. Rev. Lett. 2009, 102, 073005 10.1103/PhysRevLett.102.073005. 19257665

[ref35] Parker V. D. Energetics of Electrode Reactions. II. The Relationship between Redox Potentials, Ionization Potentials, Electron Affinities, and Solvation Energies of Aromatic Hydrocarbons. J. Am. Chem. Soc. 1976, 98, 98–103. 10.1021/ja00417a017.

[ref36] Matsumoto A.; Suzuki M.; Hayashi H.; Kuzuhara D.; Yuasa J.; Kawai T.; Aratani N.; Yamada H. Aromaticity Relocation in Perylene Derivatives upon Two-Electron Oxidation To Form Anthracene and Phenanthrene. Chem. - Eur. J. 2016, 22, 14462–14466. 10.1002/chem.201602188. 27429200

[ref37] Elgrishi N.; Rountree K. J.; McCarthy B. D.; Rountree E. S.; Eisenhart T. T.; Dempsey J. L. A Practical Beginner’s Guide to Cyclic Voltammetry. J. Chem. Educ. 2018, 95, 197–206. 10.1021/acs.jchemed.7b00361.

[ref38] Wu Z.; Zhang Z.; Liu L. Electrochemical Studies of a Cu(II)-Cu(III) Couple: Cyclic Voltammetry and Chronoamperometry in a Strong Alkaline Medium and in the Presence of Periodate Anions. Electrochim. Acta 1997, 42, 2719–2723. 10.1016/S0013-4686(97)00015-7.

[ref39] Gouré E.; Gerey B.; Molton F.; Pécaut J.; Clérac R.; Thomas F.; Fortage J.; Collomb M.-N. Seven Reversible Redox Processes in a Self-Assembled Cobalt Pentanuclear Bis(Triple-Stranded Helicate): Structural, Spectroscopic, and Magnetic Characterizations in the Co I Co II 4, Co II 5, and Co II 3 Co III 2 Redox States. Inorg. Chem. 2020, 59, 9196–9205. 10.1021/acs.inorgchem.0c01102. 32579848

