# Supplementary material for: Perylene-Based Coordination Polymers: Synthesis, Fluorescent J-Aggregates, and Electrochemical Properties
Source: Inorg Chem. 2023 May 8;62(20):7834–42. doi: 10.1021/acs.inorgchem.3c00540 (PMC10207335; doi:10.1021/acs.inorgchem.3c00540)
Supplement: Supplementary file 1 — ic3c00540_si_001.pdf [file ic3c00540_si_001.pdf]

Supporting Information

Perylene-based Coordination Polymers: Synthesis, Fluorescent J-aggregates, and Electrochemical Properties

Gonçalo Valente,[†] María Esteve-Rochina,[‡] Sergio P. C. Alves,[§] José M. G. Martinho,[§] Enrique Ortí,[‡] Joaquín Calbo,[‡] Filipe A. Almeida Paz,[†] João Rocha,[†] Manuel Souto[†,*]

[†] Department of Chemistry, CICECO-Aveiro Institute of Materials, University of Aveiro, Aveiro, 3810-393, Portugal

[‡] Instituto de Ciencia Molecular (ICMol), Universidad de Valencia, c/Catedrático José Beltrán, 2, 46980 Paterna, Spain

[§] Centro de Química-Física Molecular e Instituto de Nanociência e Nanotecnologia, Instituto Superior Técnico, Universidade de Lisboa, 1049-001 Lisboa, Portugal

Corresponding Author: Manuel Souto - https://orcid.org/0000-0003-3491-6984; e-mail: manuel.souto@ua.pt

Contents

1. **General methods and materials**
2. **Synthesis and characterization of the H_4PTCA ligand**
3. **Synthesis of PTC-TM MOFs**
4. **Characterization of PTC-TM MOFs**
   - **4.1. FT-IR**
   - **4.2. PXRD and Rietveld refinement**
   - **4.3. Solid-State NMR**
   - **4.4. TGA**
   - **4.5. SEM and EDS**
   - **4.6. Optical properties**
   - **4.7. Theoretical calculations**
   - **4.8. Solid-state cyclic voltammetry**
5. **References**

1. General methods and materials

All reagents and solvents employed in the syntheses were of high purity grade and were purchased from Sigma-Aldrich Co. and TCI. ^{1}H liquid-state NMR spectra were recorded on a Bruker AVANCE 300 spectrometer (300 MHz). Dimethylsulfoxide-d_6 (DMSO-d_6) was used as solvent. Tetramethylsilane (TMS) was used as internal reference. Chemical shifts (δ) are quoted in ppm from TMS and the coupling constants (J) are given in Hz. ^{1}H and ^{13}C solid-state NMR spectra were recorded on a 9.4 T Bruker Avance III 400 spectrometer using a 4 mm Bruker magic-angle spinning (MAS) probe. Chemical shifts are quoted in ppm from TMS using as secondary references solid adamantane. Infrared spectra were recorded in an ATR FT-IR GALAXY SERIES FT-IR 7000 (Mattson Instruments) spectrometer in the 4000–400 cm^{-1} range using powdered samples. Raman spectra were recorded in a RFS 100/S (Bruker) spectrometer equipped with Nd:YAG laser (1064 nm). Thermogravimetric analysis (TGA) was carried out with a Shimadzu TGA 50 equipment in the 25–600 ºC temperature range under a 5 ºC min^{-1} scan rate and a N_2 flow of 20 mL·min^{-1}. Powder X-ray diffraction patterns were recorded using an Empyrean PANalytical diffractometer (Cu $K\alpha_{1,2}$ X-radiation, λ_1 = 1.540598 Å; λ_2 = 1.544426 Å), equipped with an PIXcel 1D detector and a flat-plate sample holder in a Bragg-Brentano para-focusing optics configuration (45 kV, 40 mA).

The UV-vis absorption spectra were measured with a Jasco UV-660 Spectrophotometer (Jasco International, Tokyo). Fluorescence spectra were recorded with a Fluorolog 3-22 Spectrofluorimeter (Horiba Jobin Yvon, USA) with a 450 W xenon lamp. The fluorescence decay curves were recorded by the single-photon timing technique, by excitation at 285nm and 570 nm, using the vertical polarized light of the second harmonic of a Coherent Radiation Dye laser 700 series (laser dye DCM, 610–680 nm, 130 mW, 5 ps, 4 MHz). The emission was collected at the magic angle using a Jobin Yvon HR320 monochromator (Horiba Jovin Ivon Inc.). The instrument response functions (35–80 ps FWHM) were generated by the scattering of colloidal silica water dispersions. Decay curves were stored in 1024 channels with an accumulation of at least 20k counts in the peak channel. The photoluminescence decay curves were fitted by a non-linear least-squares reconvolution method using the TRFA DP software by SSTC (Scientific Software Technologies Center, Belarusian State University, Minsk, Belarus).[1]

2. Synthesis of the H_4PTCA ligand

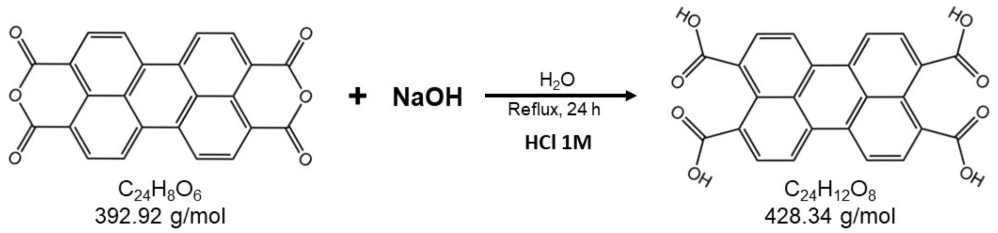

Scheme S1. Synthesis of the 3,4,9,10-perylenetetracarboxylic acid (H_4PTCA).

The ligand 3,4,9,10-perylenetetracarboxylic acid (H_4PTCA) was synthesized by reacting 3,4,9,10-perylenetetracaboxylicdianhydride (PTCDA) (500 mg, 1,27 mmol) and sodium hydroxide (800 mg, 20 mmol) in 20 mL of distilled water with vigorous stirring under reflux during 24 h. The resulting solution was acidified with 1 M HCl (30 mL) yielding a red precipitate. The precipitate was filtered and washed with water under reduced pressure several times. The resulting red powder was dried at 120 ºC to give 400 mg of pure ligand H_4PTCA (yield = 73%). IR (cm^{-1}): 3000 (broad, OH), 1680 (s, C=O), 1587, 1516, 1435, 1394, 1370, 1274, 1217, 1190, 1025, 938, 848, 804, 754, 729, 638, 599, 569, 506. ^{1}H-NMR (300 MHz, DMSO-d_6): (δ/ppm) 13.05 (s, 4H, COOH), 8.58 (d, 4H, Ar–H, J = 8.1 Hz), 8.01 (d, 4H, Ar–H, J = 7.9 Hz).

NMR of H_4PTCA

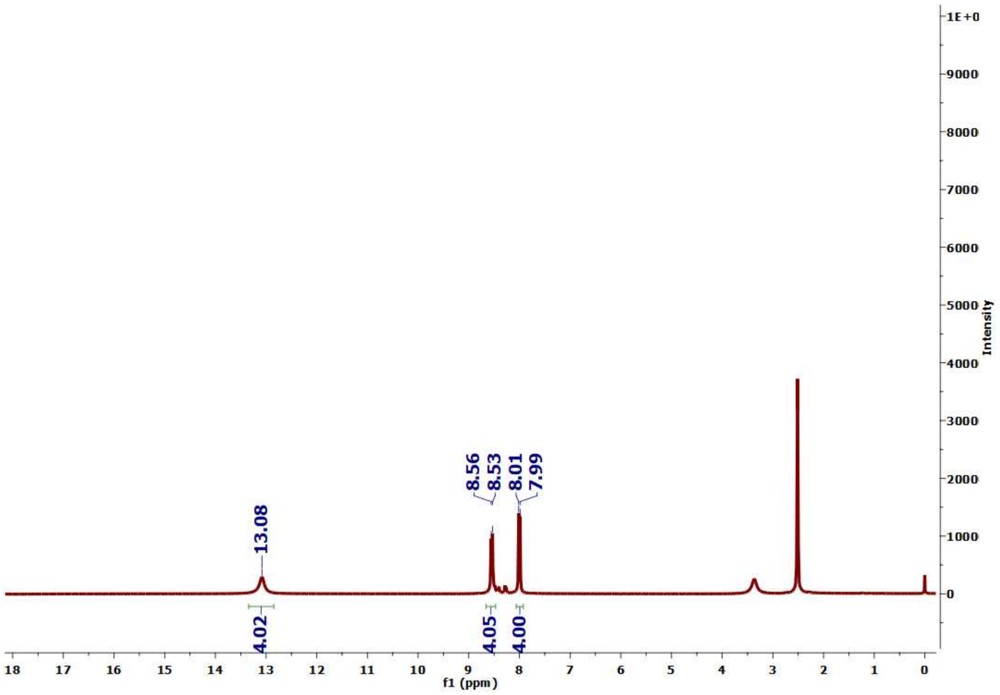

Figure S1. ^{1}H-NMR spectrum of H_4PTCA in DMSO-d_6.

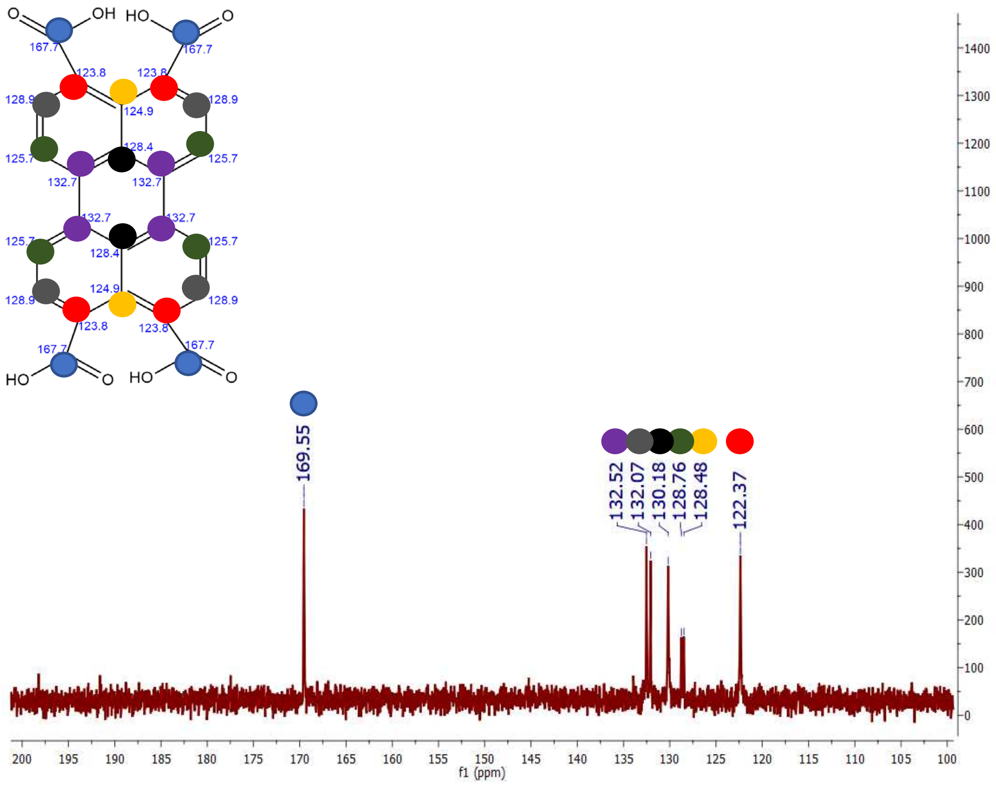

Figure S2. ^{13}C-NMR spectrum of H_4PTCA in DMSO-d_6.

Synthesis of PTC-TM MOFs

PTC-Co MOF was obtained by mixing 10 mg of 3H_4PTCA (0.023 mmol), 15 mg of $Co(CH_3CO_2)_2 \cdot 4H_2O$ (0.060 mmol), and 2 mL of EtOH/H_2O (1:3) in a Pyrex tube. The reaction mixture was sonicated for 30 minutes, and heated at 120 ºC for 5 days (↑ +0.5 ºC min^{-1}, ↓ –0.5 ºC min^{-1}). Finally, the yellow powder was filtered and washed with DMF and EtOH to obtain 10 mg of PTC-Co MOF (yield = 74 %). Elemental analysis for $Co_2C_{24}H_8O_8(H_2O)_2$: Theor: C, 49.85; H, 2.09. Exp: C, 48.50; H, 2.1.

PTC-Ni MOF was obtained by mixing 10 mg of H_4PTCA (0.0233 mmol), 15 mg of $Ni(CH_3CO_2)_2 \cdot 4H_2O$ (0.060 mmol), and 2 mL of EtOH/H_2O (1:3) in a Pyrex tube. The reaction mixture was sonicated for 30 minutes, and heated at 120 ºC for 5 days (↑ +0.5 ºC min^{-1}, ↓ -0.5 ºC min^{-1}). Finally, the light orange powder was filtered and washed with DMF and EtOH to obtain 10.2 mg of PTC-Ni MOF (yield = 76 %). Elemental analysis for Ni_2 $C_{24}H_8O_8(H_2O)_2$: Theor: C, 49.90; H, 2.09. Exp: C, 47.50; H, 2.2.

PTC-Zn MOF was obtained by mixing 10 mg of H_4PTCA (0.0233 mmol), 13 mg of $Zn(CH_3CO_2)_2 \cdot 2H_2O$ (0.058 mmol), and 2 mL of EtOH/H_2O (1:3) in a Pyrex tube. The reaction mixture was sonicated for 30 minutes, and heated at 120 ºC for 5 days (↑ +0.5 ºC min^{-1}, ↓ -0.5 ºC min^{-1}). Finally, the dark orange powder was filtered and washed with DMF and EtOH to obtain 8.3 mg of PTC-Zn MOF (yield = 72 %). Elemental analysis for $Zn_2C_{24}H_8O_8(H_2O)_2$: Theor: C, 48.77; H, 2.05. Exp: 49.05; H, 2.3.

3. Characterization of PTC-TM MOFs

3.1. FT-IR spectra

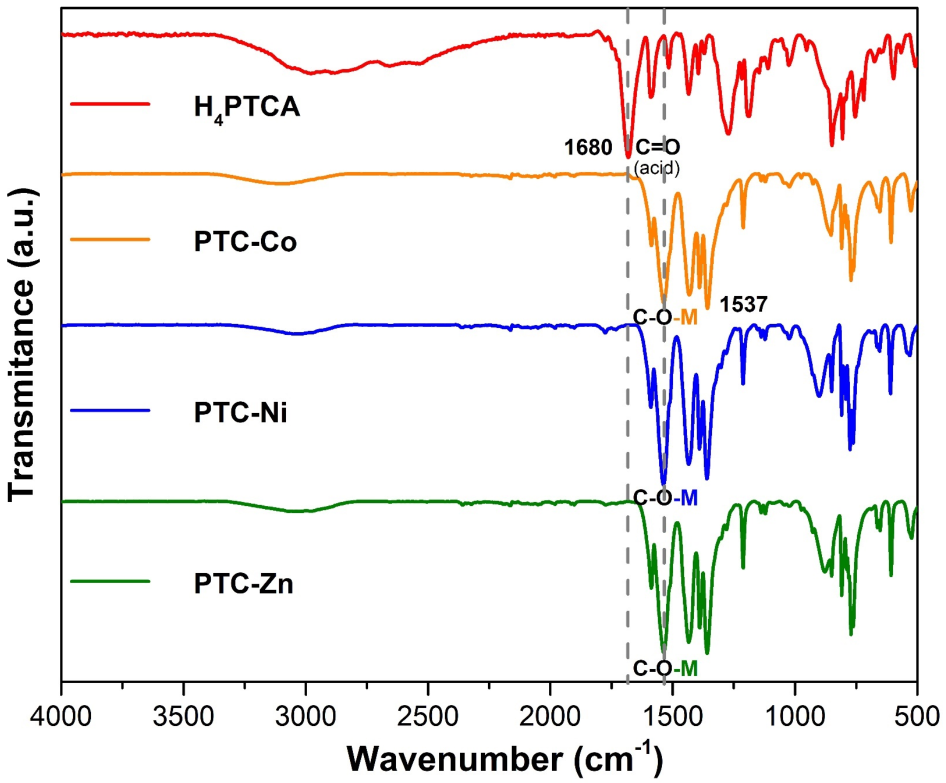

Figure S3. FT-IR spectra of **H_4PTCA**, **PTC-Co**, **PTC-Ni**, and **PTC-Zn CPs**.

3.2. Powder X-ray Diffraction and Rietveld refinement

Powder X-Ray Diffraction (PXRD) data for all compounds were collected at ambient temperature on an Empyrean PANalytical diffractometer, with a working wavelength of λ_1 = 1.540598 Å and λ_2 = 1.544426 Å (Cu K$\alpha_{1,2}$ X-radiation), equipped with a PIXcel 1D detector, a capillary sample holder, and an Incident beam PreFIX module with elliptical X-ray mirror for Cu radiation (45 kV, 40 mA). Intensity data were collected by the step-counting method, in continuous mode, in the *ca.* 4.0º ≤ 2θ ≤ 50º range.

A fine powdered sample was placed inside a Hilgenberg borosilicate glass capillary (*ca.* 1.0 mm of diameter) which was spun during data collection to improve powder averaging over the individual crystallites, ultimately removing eventual textural effects such as preferential orientation.

The collected powder X-ray diffraction patterns were indexed using the LSI-Index algorithm implemented in TOPAS-Academic V5,[2-3] and a whole-powder-pattern Pawley fit permitted to unequivocally confirm the orthorhombic *Pbam* space group as the most suitable for the compound, in good agreement with the reported crystal structure for isotypical materials.[4]

The crystal structures were also determined in TOPAS-Academic V5[2] by using a simulated annealing approach. In a first stage of the crystal solution, the metallic centers and the coordinated water molecules were allowed to converge to their optimal positions within the unit cell while using a battery of anti-bump restraints to ensure chemically reasonable coordination geometries. Despite the good quality of the collected patterns, the location of the atoms composing the crystallographically independent organic component proved to be of extreme difficulty, even when using distance restraints just like those employed for the inorganic backbone. The derivation of the most suitable location for the organic linker was performed in a second stage by using a Fenske-Hall *Z*-matrix for half of this chemical entity and treating the ligand as a rigid body inside the unit cell. We note that this strategy greatly facilitates the mobility of this chemical entity inside the unit cell boundaries during the global optimization processes. It does not, however, take into account the conformational flexibility associated with the mutual rotations of pendant moieties. This was taken into consideration by comparing the structure with the data previously published.

A Rietveld structural refinement[5] was performed with TOPAS-Academic V5[2] using either a Chebychev polynomial throughout the entire angular range to model the background contribution, or fixed background points. The peak shapes for the powder patterns were

described using the fundamental parameters approach,[6] with preferential orientation effects being modelled using a 4th order spherical harmonics approach.

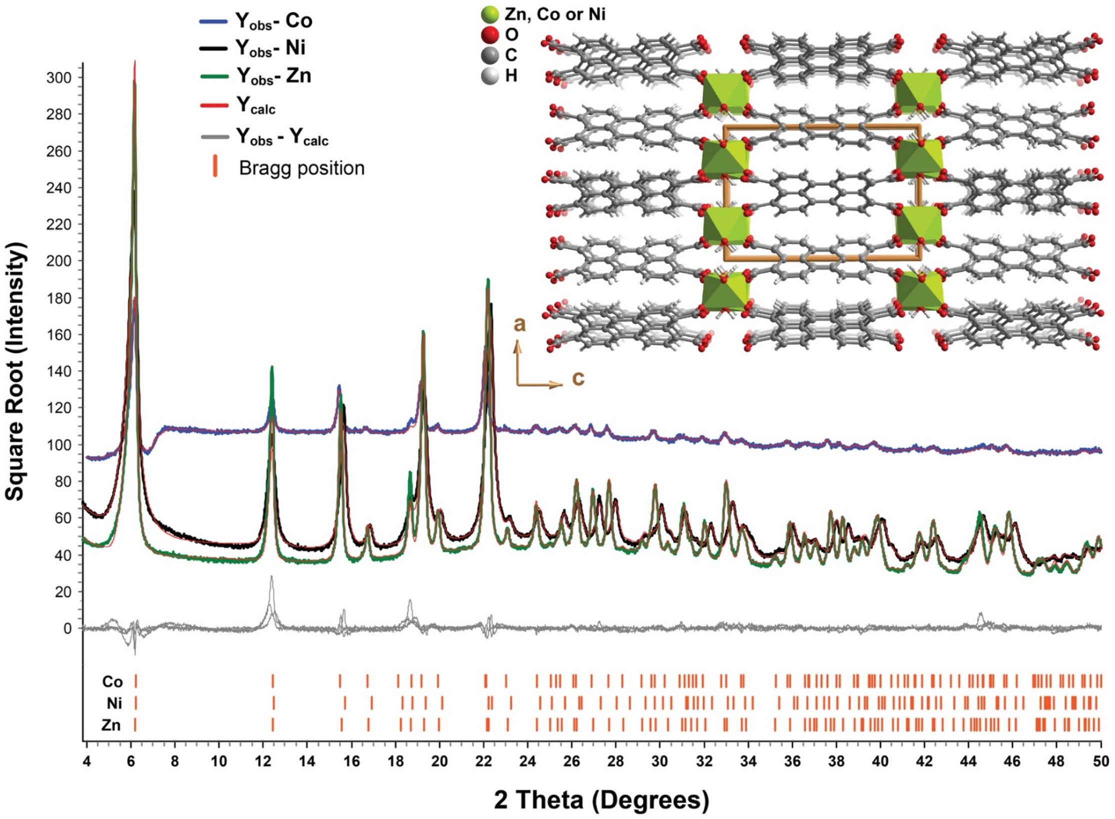

Figure S4. Final Rietveld plots of **PTC-Co**, **PTC-Ni**, and **PTC-Zn**. The inset depicts the crystal packing of the crystal structures viewed in perspective along the [010] direction of the unit cell.

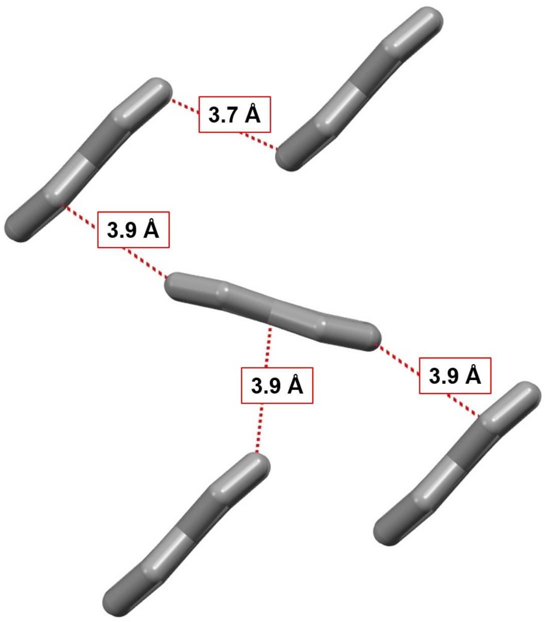

Figure S5. Partial view of the herringbone arrangement of the perylene-based linkers showing the shortest C···C distances between neighbouring perylenes in the **PTC-Zn** MOF. For simplicity, carboxylate groups and Zn atoms are omitted.

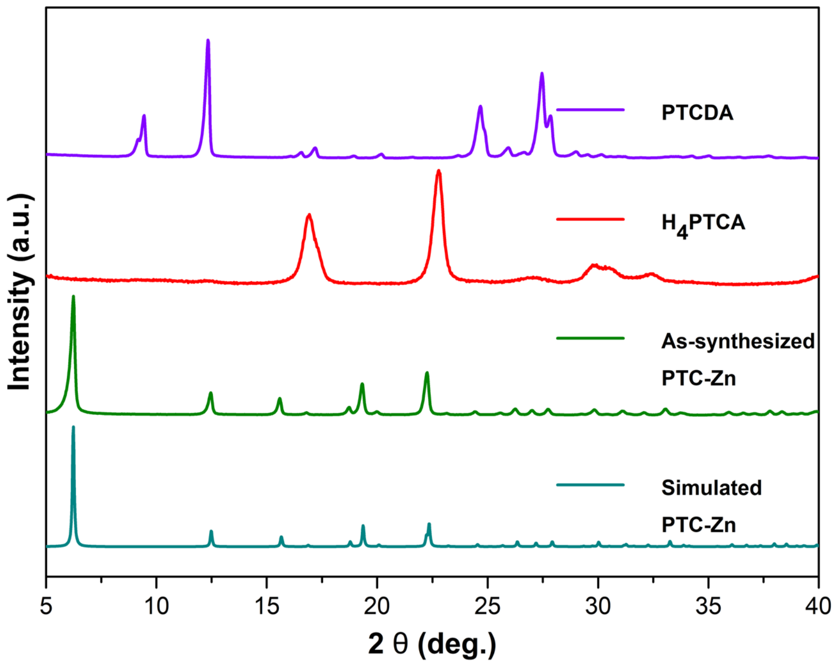

Figure S6. Powder X-ray diffraction patterns of simulated **PTC-Zn** MOF, as-synthesized **PTC-Zn** MOF, ligand H_4PTCA, and PTCDA.

3.3. ^{1}H Solid-State MAS NMR

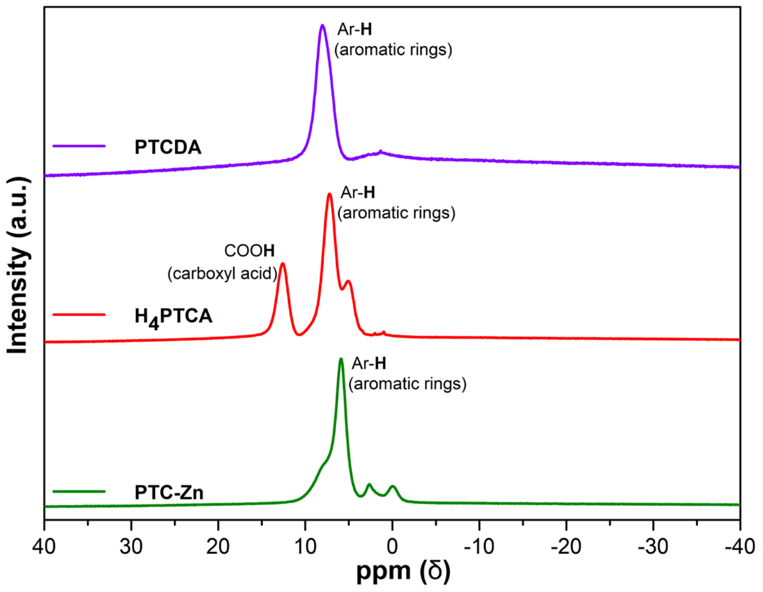

Figure S7. ^{1}H MAS NMR spectra of **PTC-Zn** MOF, H_4PTCA, and PTCDA recorded at room temperature with a spinning rate of 65 kHz.

3.4. TGA

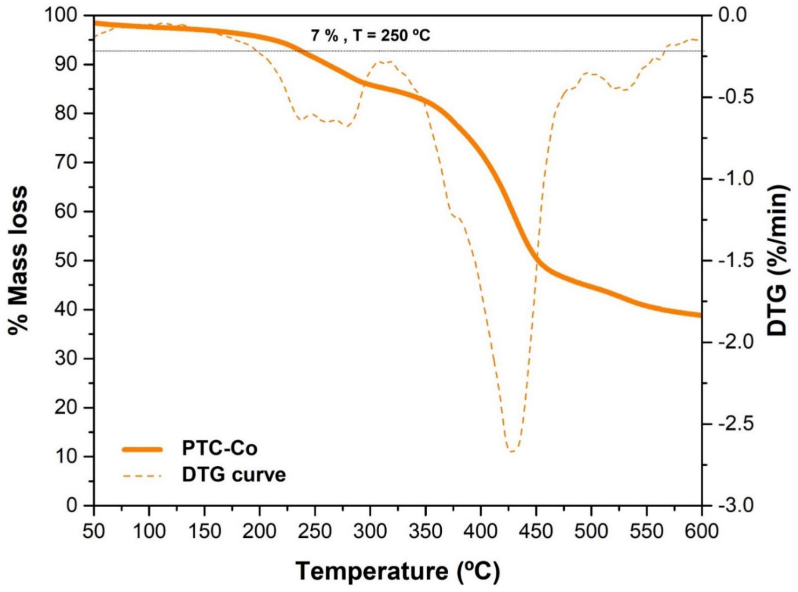

Figure S8. TGA trace (orange line) recorded for the **PTC-Co** MOF at a heating rate of 5ºC min^{-1} under a constant stream of N_2. The first derivative of the TGA trace, the DTG curve dashed orange line), is also shown.

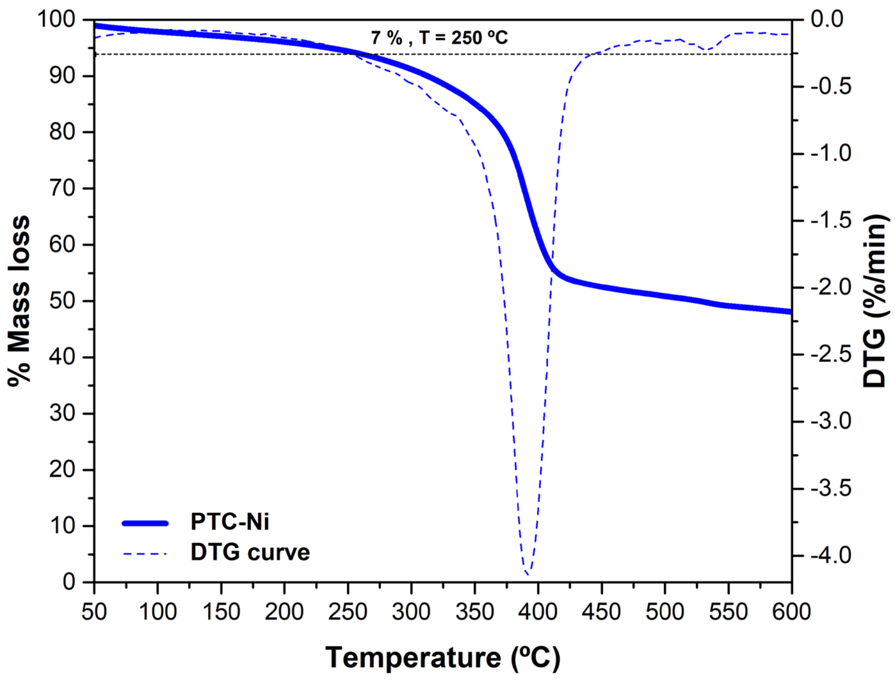

Figure S9. TGA trace (blue line) recorded for the **PTC-Ni** MOF at a heating rate of 5ºC min^{-1} under a constant stream of N_2. The DTG curve (dashed blue line) is also shown.

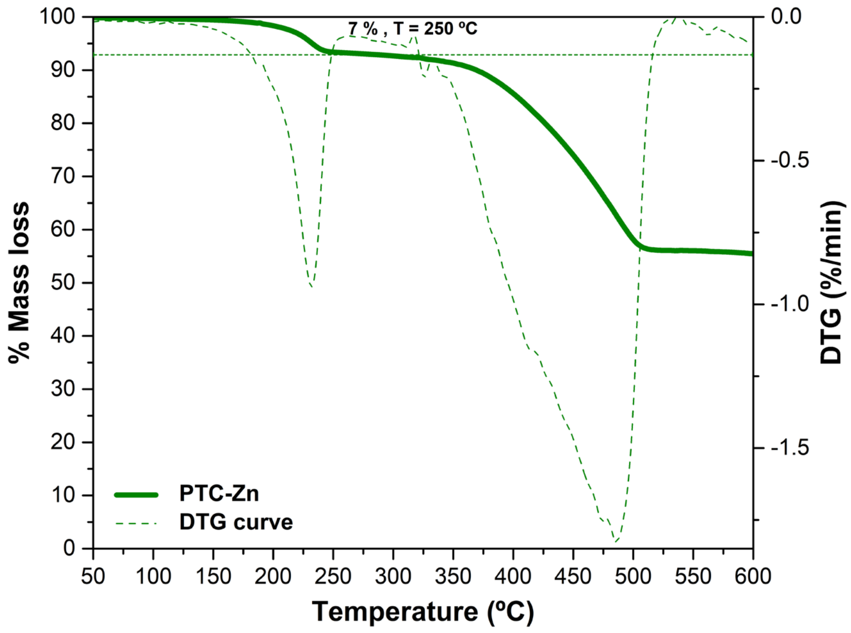

Figure S10. TGA trace (green line) recorded for the **PTC-Zn** MOF at a heating rate of 5ºC min^{-1} under a constant stream of N_2. The DTG curve (dashed green line) is also shown.

3.5. SEM and EDS Mapping

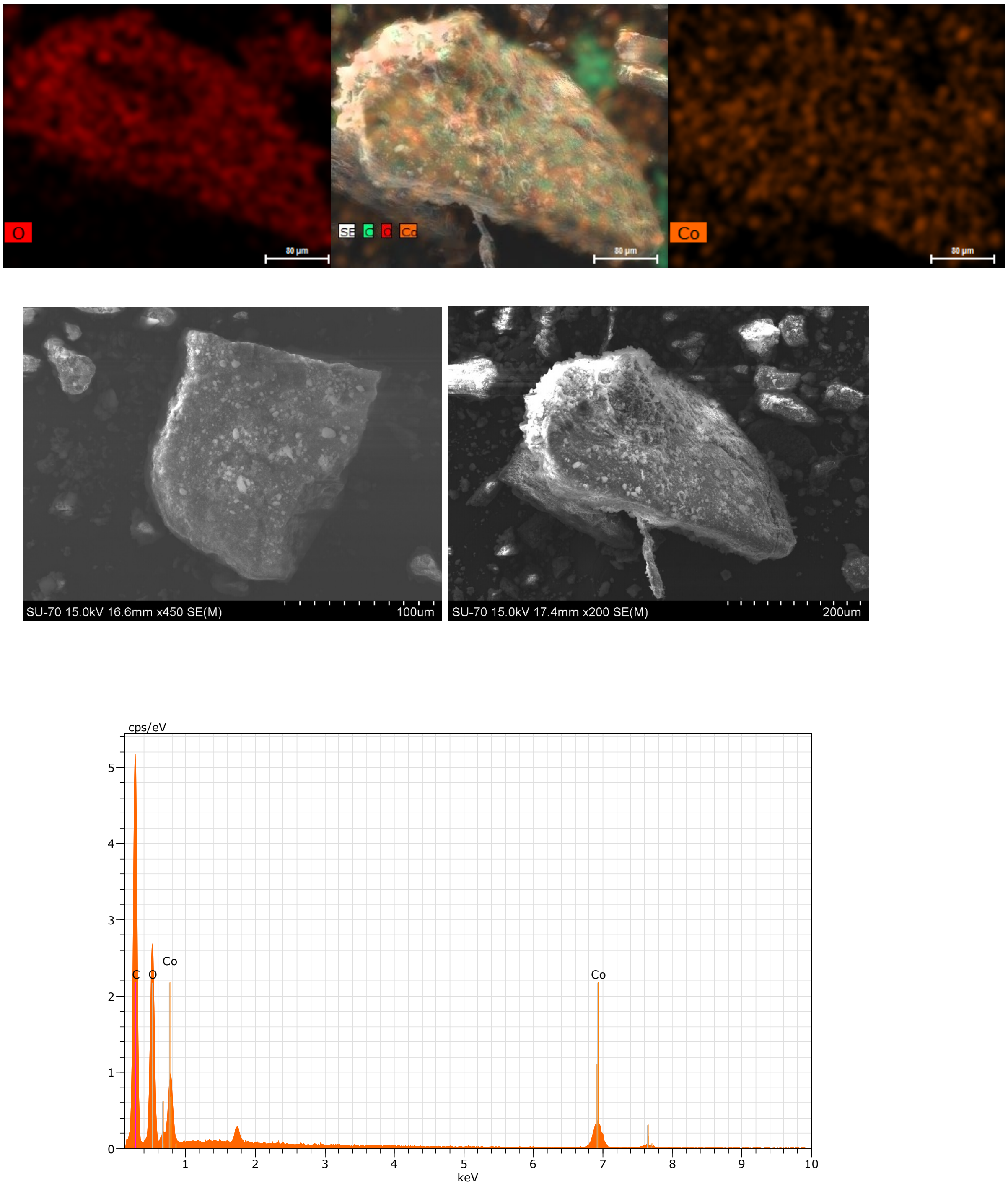

Figure S11. SEM images (top) and energy dispersive X-ray spectroscopy (EDS, bottom) recorded for the **PTC-Co** MOF.

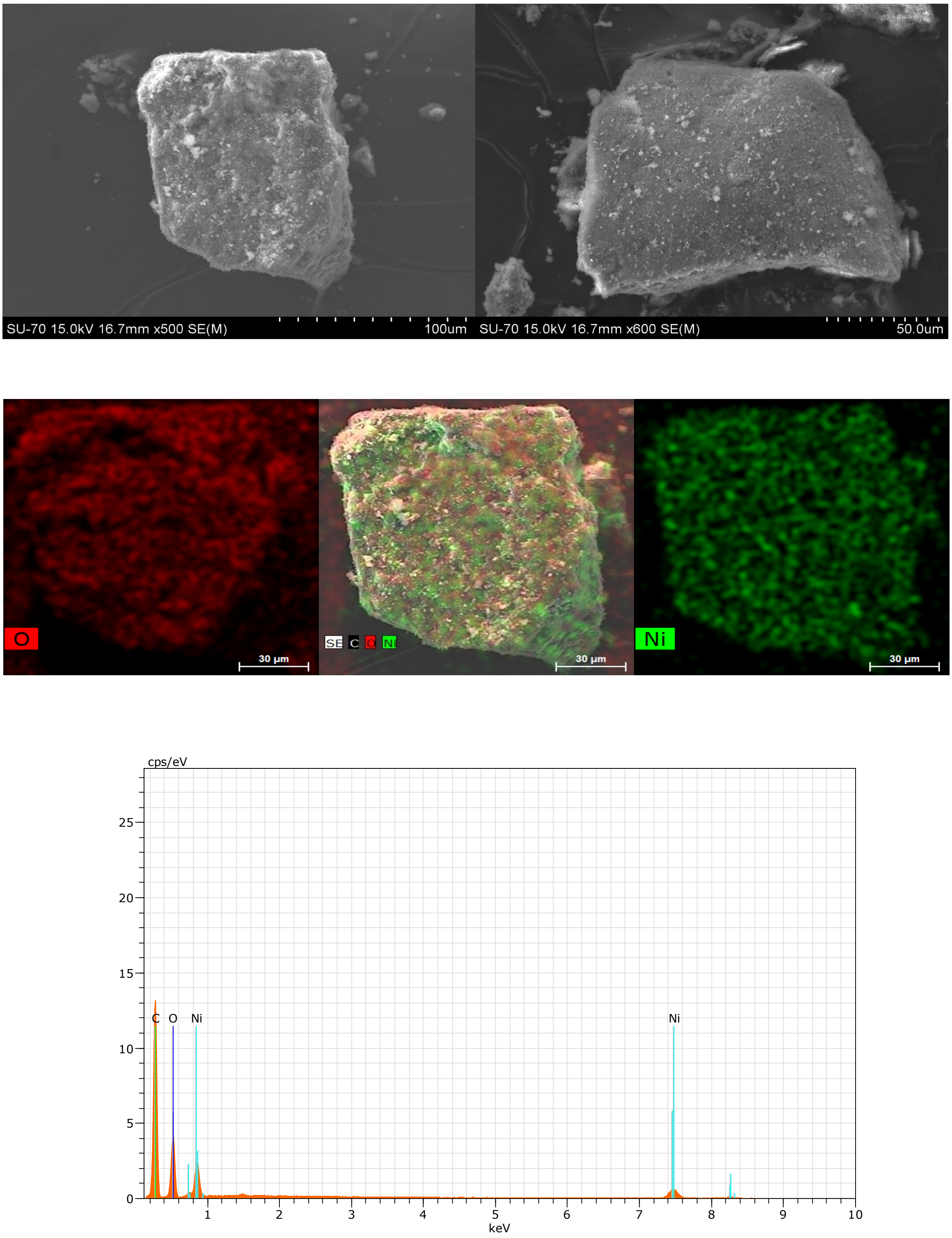

Figure S12. SEM images and energy dispersive X-ray spectroscopy (EDS) recorded for the **PTC-Ni** MOF.

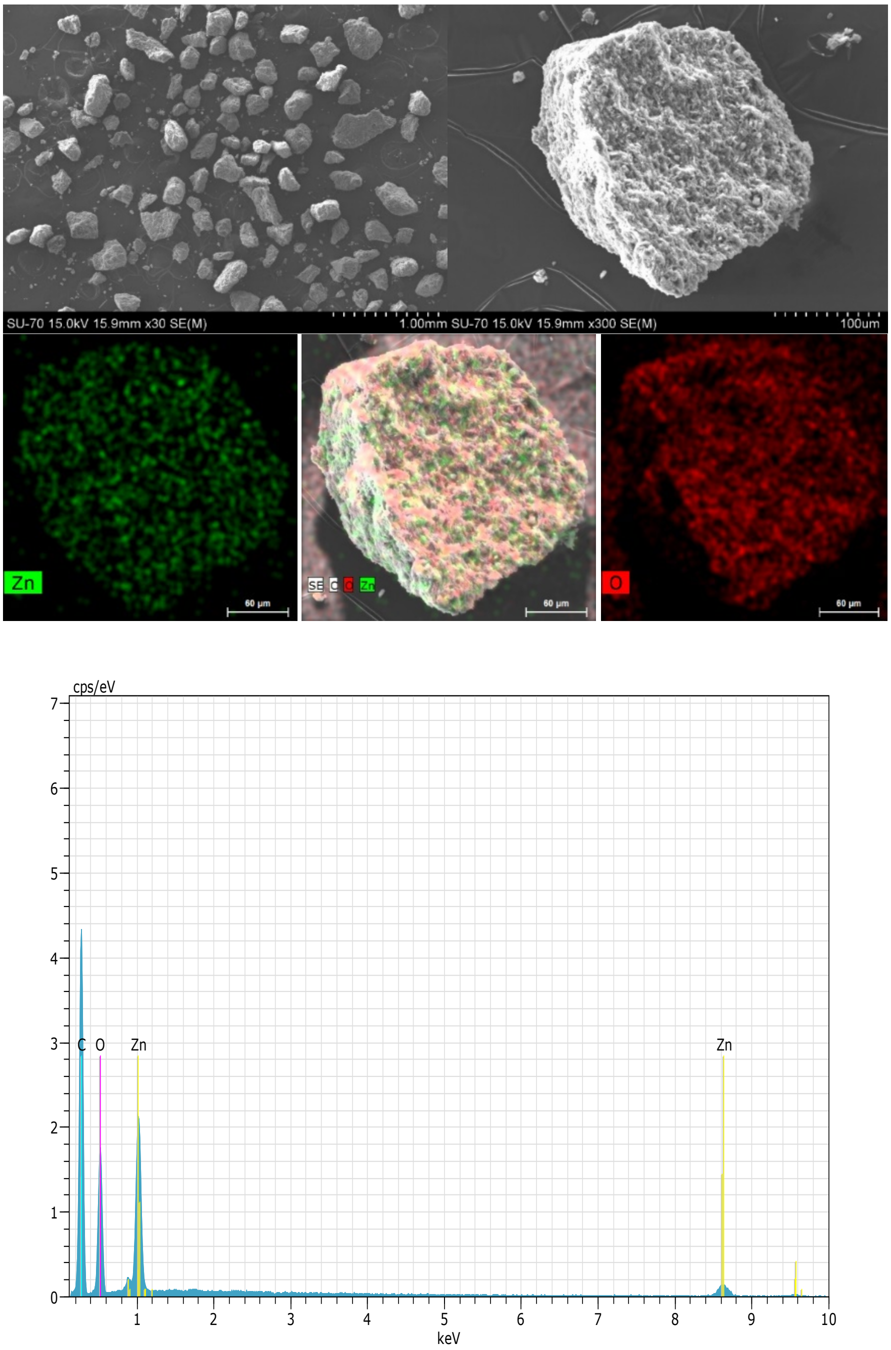

Figure S13. SEM images and energy dispersive X-ray spectroscopy (EDS) recorded for the **PTC-Zn** MOF.

3.6. Optical properties

UV/visible absorption and diffuse reflectance: The UV-vis-NIR absorption and diffuse reflectance spectra of the samples were measured using a Lambda 950 dual-beam spectrometer (PerkinElmer) and Reflectance FLEX Pack (Sarspec). The diffuse reflectance spectra are reported as the Kubelka-Munk transform, where $F(R) = (1-R)^2/2R$. The direct optical band gaps of these materials were determined from respective Tauc plots.

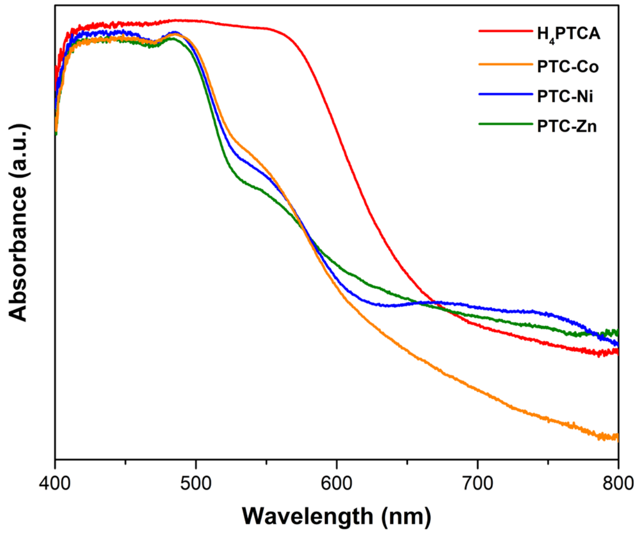

Figure S14. Solid-state UV-vis-NIR absorbance spectra for **PTC-Co**, **PTC-Ni**, and **PTC-Zn** MOFs.

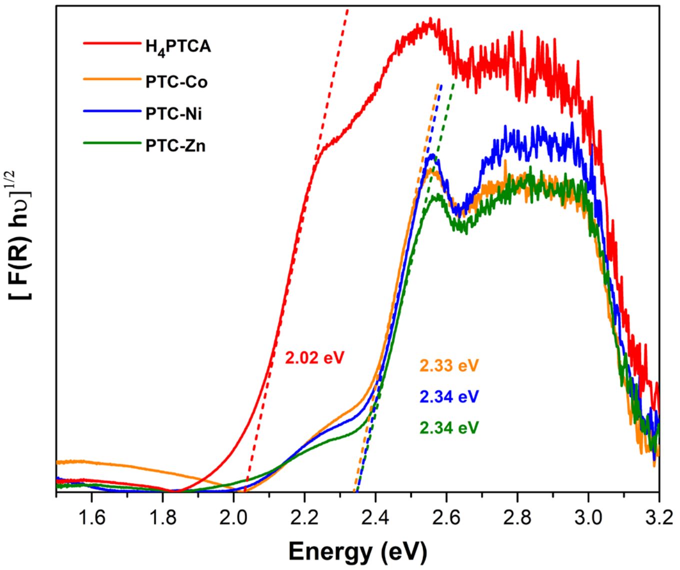

Figure S15. Diffuse UV-vis-NIR reflectance of **PTC-TM** CPs and H_4PTCA ligand. Normalized Tauc plot of the Kubelka–Munk-transformed data. Dashed lines indicate linear fits to the absorption onsets.

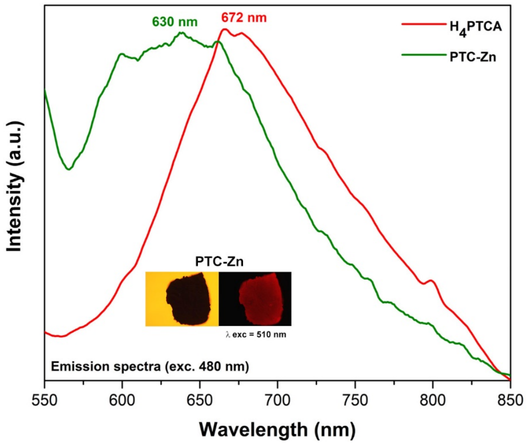

Figure S16. Emission spectra in the solid state for **H_4PTCA** and **PTC-Zn** CP with excitation at 480 nm. The inset shows the picture of PTC-Zn in solid state taken with an optical microscope under normal light and upon excitation at $\lambda_{exc} = 510$ nm.

a)

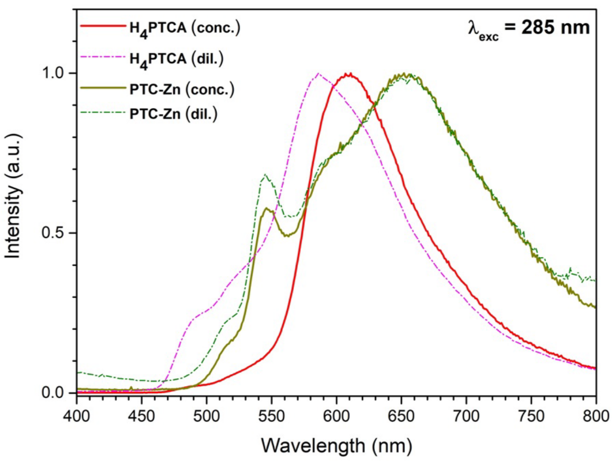

b)

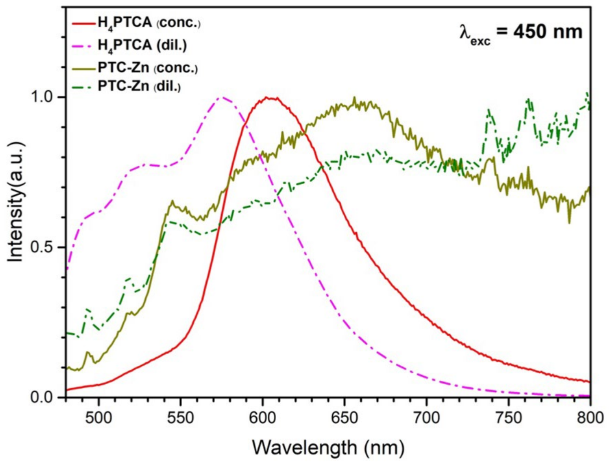

Figure S17. Emission spectra of H_4PTCA (red) and **PTC-Zn** (green) suspensions in EtOH at a) $\lambda_{exc} = 285$ nm and b) $\lambda_{exc} = 450$ nm. The concentration of the suspension is 0.2 (conc.) and 0.08 mg mL^{-1} (dil.)

a)

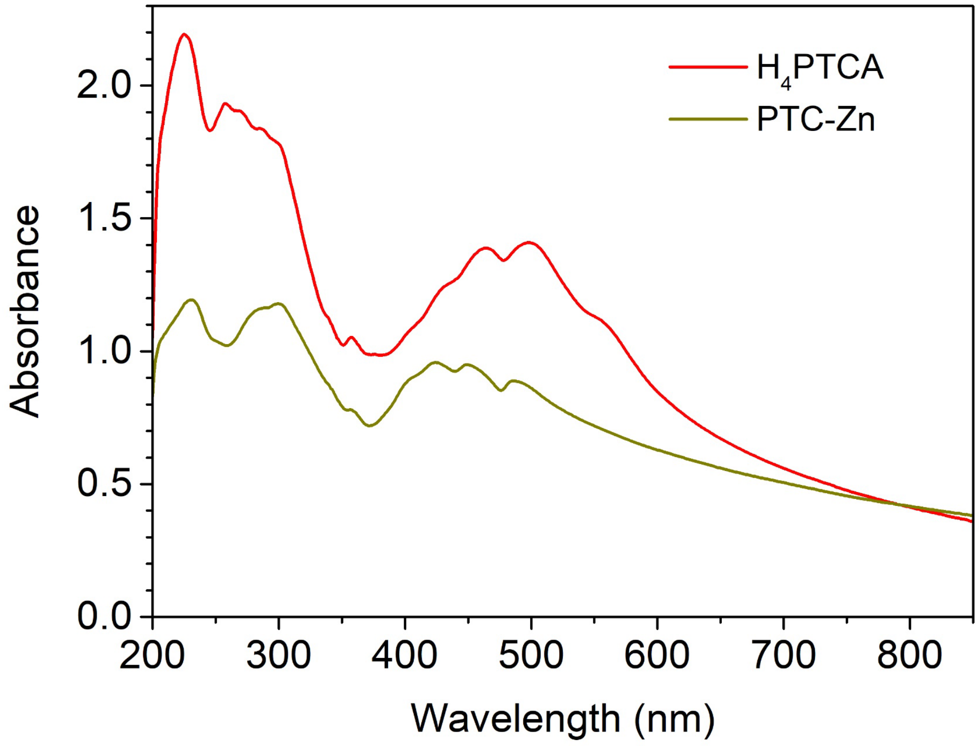

b)

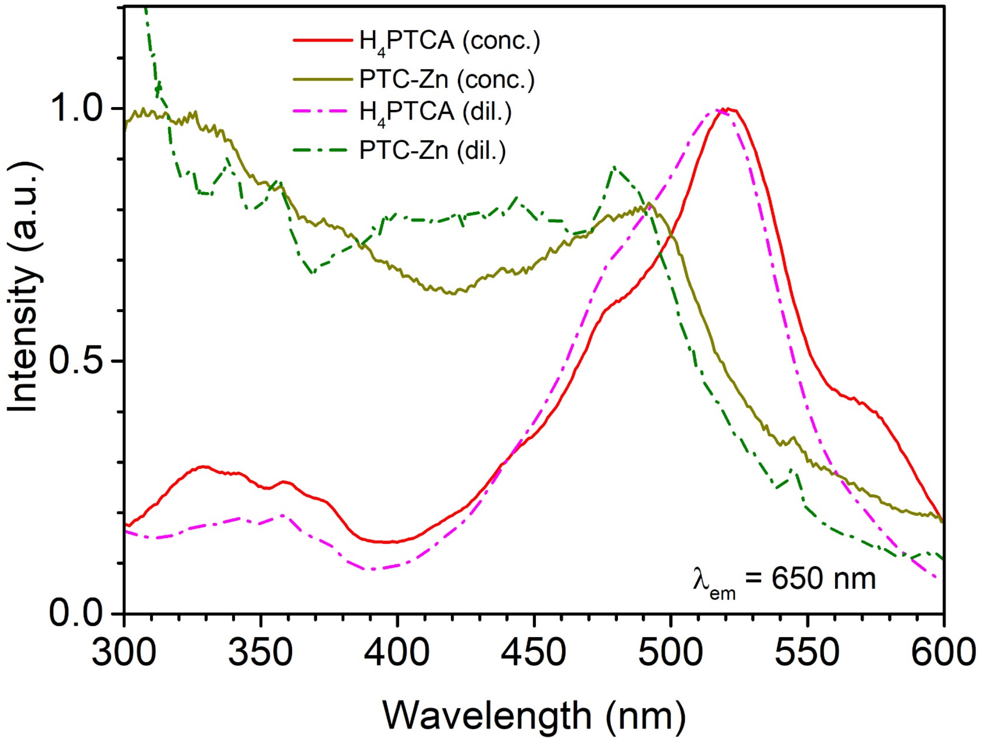

Figure S18: a) Absorption spectra of H_4PTCA (red) and **PTC-Zn** (green) suspensions in EtOH. b) Excitation spectra of H_4PTCA and **PTC-Zn** (λ_{em} = 560 nm). Concentrations of 0.2 (conc.) and 0.08 mg mL^{-1} (dil.) were used.

3.7. Theoretical calculations

Molecular calculations of H_4PTCA in its neutral form in gas phase were performed under the density functional theory (DFT) framework using the Gaussian-16.A03 suite of programs.[7] Minimum-energy structure and electronic structure calculations were obtained at the PBE0/6-31G(d,p) level of theory.[8]

Quantum-chemical calculations in periodic boundary conditions were performed within the density functional theory (DFT) framework as implemented in the all-electron full-potential FHI-AIMS electronic structure code package.[9] The minimum-energy structures of **PTC-TM** CP materials were obtained, starting from the experimental X-ray data, upon full lattice and ionic relaxation using the GGA-type PBEsol functional[10] and the numeric atom-centered orbital light tier-1 basis set. Dispersion forces were included by means of the vdW Hirshfeld correction as described by Tkatchenko and Scheffler.[11] The electronic band structure and density of states (DOS) were calculated using the hybrid HSE06 functional.[12] A full *k*-path in the *Pbam* first Brillouin zone of Γ–X–S–Y–Γ–Z–U–R–T–Z–Γ and a 3×3×3 *k*-grid were employed. Effective masses for hole and electron along the *k*-path segments in the valence band maximum (VBM) and conduction band minimum (CBM) were calculated according to the parabolic approximation by using the aims_effect_mass.py utility included in FHI-AIMS. The absorption spectra of the **PTC-TM** CPs were computed under the dielectric function approximation as implemented in FHI-AIMS, using the hybrid HSE06 functional and the light tier-1 basis set.

Time-Dependent DFT (TD-DFT) calculations[13] were performed at the PBE0/6-31G(d,p) level of theory for the lowest-lying excited states of H_4PTCA monomer and representative dimers, as extracted from the crystal structures, in gas phase by using the Gaussian-16A.03 software.[7] The minimum-energy optimized geometry of the low-lying bright state was obtained for H_4PTCA, and its harmonic frequencies were calculated at the TD-DFT/PBE0/6-31G(d,p) level of theory. Vibrational resolution of the H_4PTCA $S_0 \rightarrow S_1$ electronic transition was calculated by means of the Franck-Condon principle as implemented in Gaussian-16.A03. To simulate the charge-transfer state and compute the excimer-like band energy, the internal structure of the PTC ligands in the dimers was replaced by the minimum-energy structure of a PTC cation and a PTC anion, maintaining the intermolecular disposition unchanged.

The excitonic coupling between the PTC ligands in the MOF was estimated for the three representative dimers (A, B, and C, Figure 7 in the main text) extracted from the minimum-energy crystal structure of **PTC-Zn, PTC-Ni**, and **PTC-Co**, and using the electronic energy transfer (EET) analysis as coded in Gaussian-16.A03.[7]

The crystalline geometries, spin densities, and frontier crystal orbitals were displayed using the software VESTA.[14] The molecular orbital topologies were plotted by means of the Chemcraft software.[15]

Table S1. Relative energy of the different spin configurations optimized at the PBEsol/light tier1 level and single-point calculated at the HSE06/light tier 1 level for the **PTC-TM** materials. FM, AFM, LS, and HS stem from ferromagnetic, antiferromagnetic, low spin, and high spin, respectively. Two AFM configurations are calculated in each case: config. 1 with the closest TM atoms arranged in an antiparallel configuration, and config. 2 with the farthest TM atoms arranged in antiparallel.

**Spin configuration**	**Relative energy (eV)**
**Ni(II) FM**	0.00
Ni(II) AFM config. 1	0.34
Ni(II) AFM config. 2	0.01
**Co(II) HS FM**	0.00
Co(II) HS AFM config. 1	5.07
Co(II) HS AFM config. 2	0.26
Co(II) LS FM	3.27
Co(II) LS AFM config. 1	3.19
Co(II) LS AFM config. 2	3.28

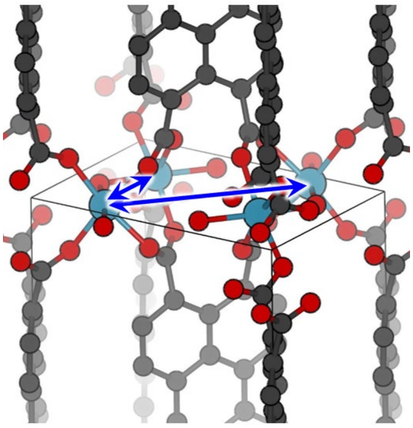

Table S2. TM···O distances (in Å) and coordinated water tilting from perpendicular direction with respect to the TM–O(carboxylate) plane (in º), calculated for the quasi-octahedral metal coordination environment in **PTC-TM** crystals.

**System**	**TM···O(carboxylate)**	**TM···O(water)**	**water tilting**
**PTC-Zn**	2.10	2.07	2
	2.07	2.09	9
	2.10	-	-
	2.07	-	-
**PTC-Ni**	2.04	2.05	9
	2.01	2.01	8
	2.04	-	-
	2.01	-	-
**PTC-Co**	2.05	2.10	10
	2.02	2.05	6
	2.05	-	-
	2.02	-	-

Table S3. Lattice parameters calculated for the theoretical minimum-energy crystal structures of **PTC-TM** CPs at the PBEsol level of theory. The experimental X-ray data are also indicated for comparison.

System	*a* (Å)	*b* (Å)	*c* (Å)	*a* (°)	*b* (°)	γ(°)	*V* (Å^3)
**PTC-Zn** (theo)	9.590	6.791	14.291	90.0	90.0	90.0	930.5
**PTC-Zn** (exp)	9.718	7.024	14.230	90.0	90.0	90.0	971.3
**PTC-Ni** (theo)	9.528	6.681	14.188	90.0	90.0	90.0	902.8
**PTC-Ni** (exp)	9.686	6.932	14.177	90.0	90.0	90.0	951.9
**PTC-Co** (theo)	9.625	6.718	14.192	90.0	90.0	90.0	917.7
**PTC-Co** (exp)	9.772	7.040	14.200	90.0	90.0	90.0	977.0

Table S4. Relevant geometrical parameters and excitonic couplings (J_{exc}) calculated for the different dimers extracted from the optimized crystal structure of **PTC-TM** CPs (Figure 7).

System	Dimer	Perylene centroid-centroid intermolecular distance (Å)	Closest intermolecular distance (Å)	J_{exc} (meV)
**PTC-Zn**	A	6.791	3.530 (π–π)	59
	B	5.876	2.798 (CH–π)	69
	C	14.291	-	–32
**PTC-Ni**	A	6.681	3.483 (π–π)	62
	B	5.817	2.728 (CH–π)	71
	C	14.291	-	–32
**PTC-Co**	A	6.718	3.500 (π–π)	62
	B	5.867	2.861 (CH–π)	71
	C	14.192	-	–33

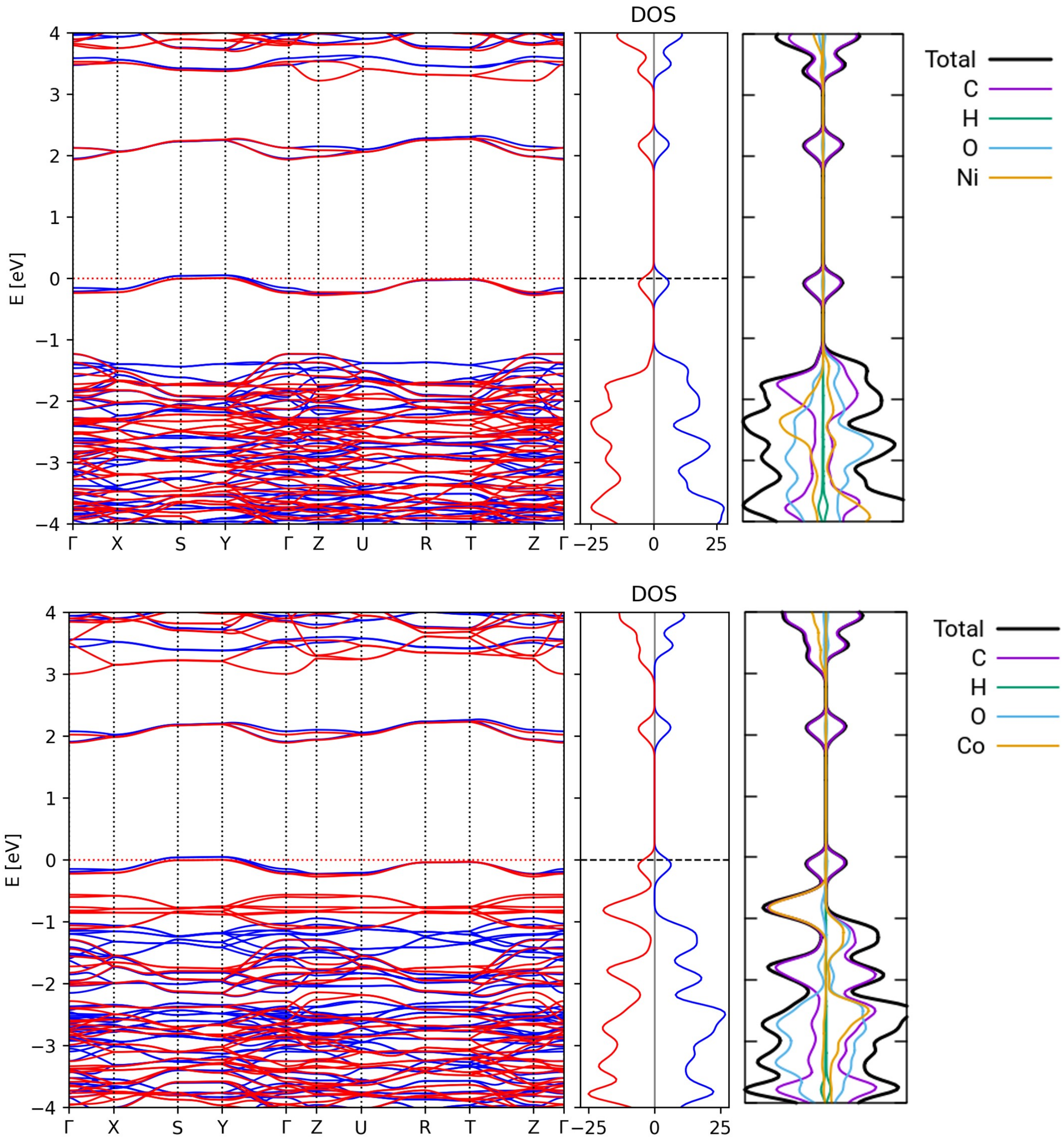

Figure S19. Band structure and density of states (DOS) calculated for **PTC-Ni** (top) and **PTC-Co** (bottom) at the HSE06/light tier-1 level of theory. Total (spin-up in blue and spin-down in red) and atom-projected contributions to the DOS are displayed. The Fermi level was set to the top of the valence band.

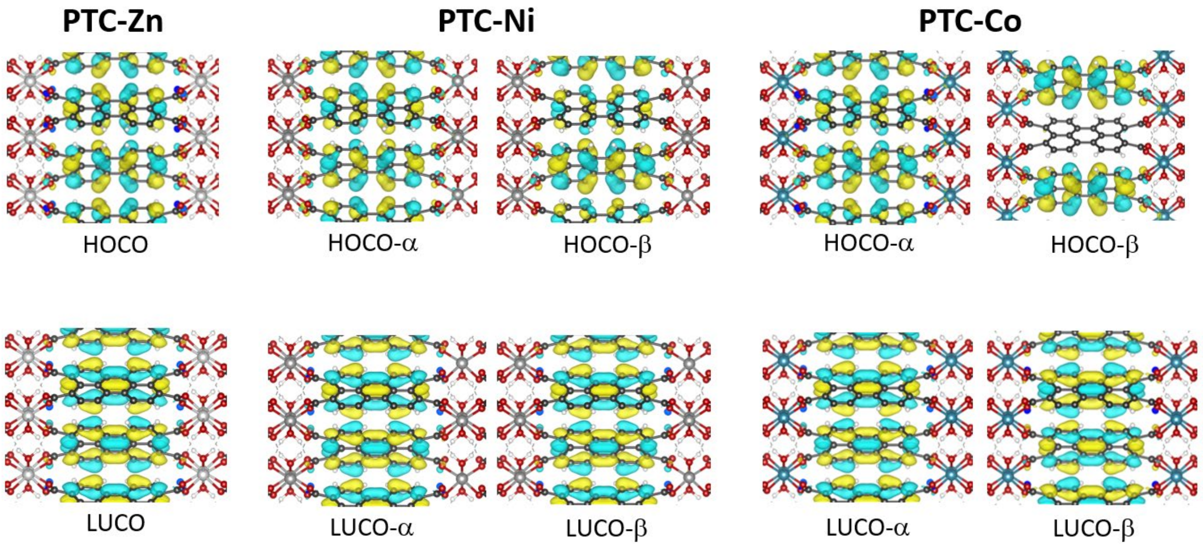

Figure S20. Frontier crystal orbitals calculated for **PTC-TM** materials at the HSE06/light tier-1 level of theory.

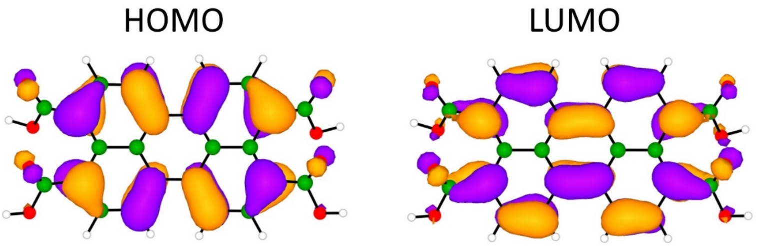

Figure S21. Highest-occupied (HOMO) and lowest-unoccupied molecular orbital (LUMO) topologies (isovalue = 0.03) calculated at the PBE0/6-31G(d,p) level of theory for the H_4PTCA molecule.

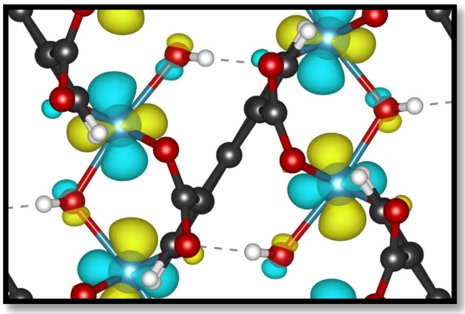

Figure S22. Crystal orbital topology corresponding to the HOCO-2(β) of **PTC-Co** calculated at the HSE06/light tier-1 level.

Table S6. Effective mass for hole (m_h^*) and electron (m_e^*) in m_0 units calculated along the full *k*-path for the VBM and CBM, respectively, in **PTC-Zn**.

	**Γ−X**	**X−S**	**S−Y**	**Y−Γ**	**Γ−Z**	**Z−U**	**U−R**	**R−T**	**T−Z**
$\boldsymbol{m_h^*}$	17.26	1.18	4.29	51.41	100.96	11.40	0.96	4.43	2.24
$\boldsymbol{m_e^*}$	6.54	1.43	2.11	7.74	383.80	4.88	0.93	1.46	2.73

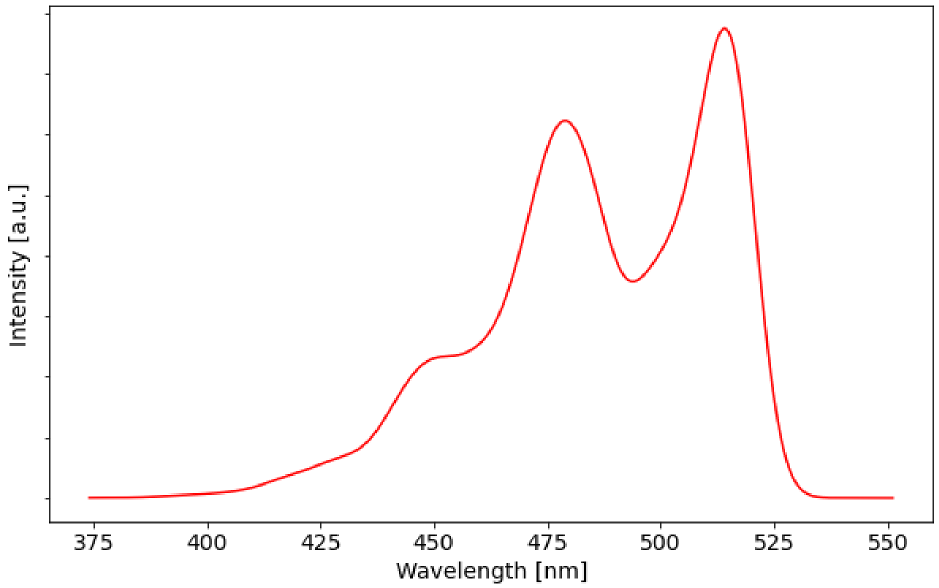

Figure S23. Vibrational resolution of the lowest-lying singlet excited state S_1 calculated for the H_4PTCA ligand at the PBE0/6-31G(d,p) level of theory.

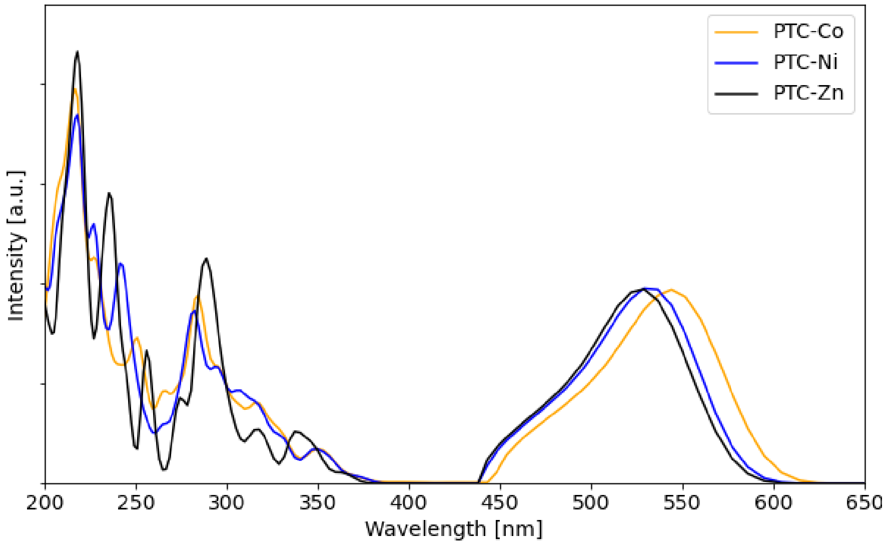

Figure S24. Simulated absorption spectra calculated for **PTC-TM** crystals via the linear macroscopic dielectric function approximation at the HSE06/light tier-1 level of theory.

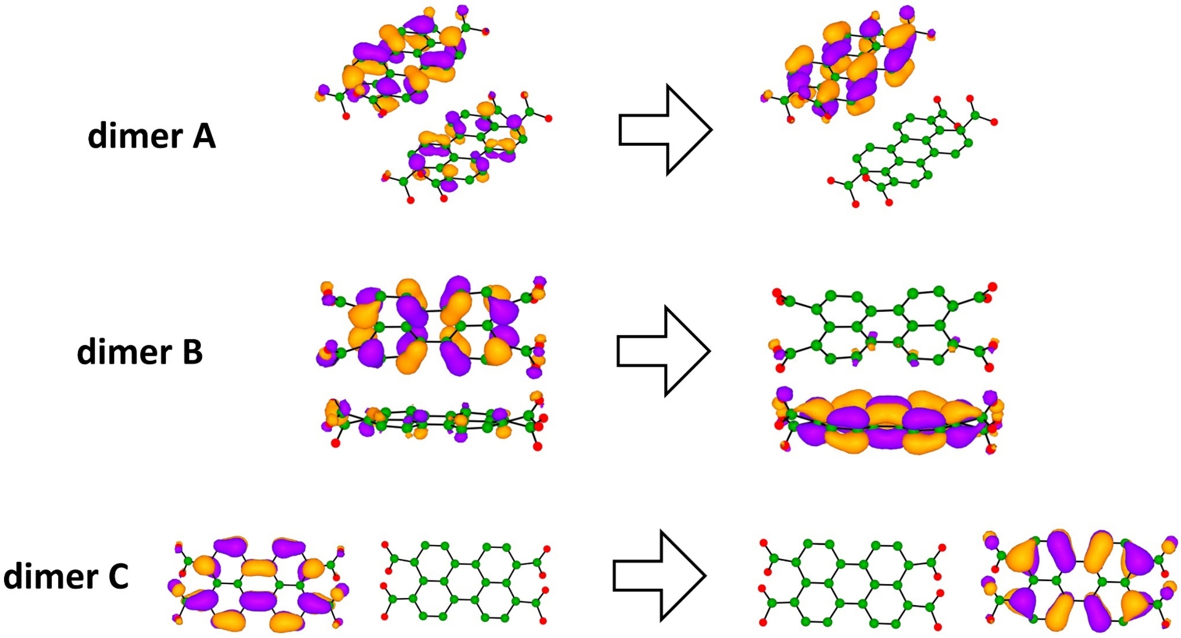

Figure S25. Monoexcitation that describes the charge transfer nature of the lowest-lying singlet excited state S_1 in dimers A, and B, and C.

3.8. Cyclic voltammetry

Electrode preparation: The powdered materials (2 mg) were mixed in 2 mL of Nafion and ethanol (1:3). 100 μL were deposited on a 3 mm diameter glassy carbon disc working electrode, which was previously polished with 0.3, 0.1, and 0.05 μm alumina powders. Afterwards, the solvent was evaporated at room temperature.

Equipment: The electrochemical experiments were performed using an Autolab electrochemical workstation (PGSTAT302N with FRA32M Module) connected to a personal computer that uses Nova 2.1 electrochemical software. A typical three-electrode experimental cell equipped with a platinum wire as the counter electrode and a silver wire as the pseudoreference electrode was used for the electrochemical characterization of the working electrodes. The electrochemical properties were studied measuring the cyclic voltammogram at different scan rates in previously N_2 purged 0.1 M $TBAPF_6/CH_3CN$ solution. Ferrocene was added as an internal standard upon completion of each experiment. All potentials are reported in V versus Ag/AgCl.

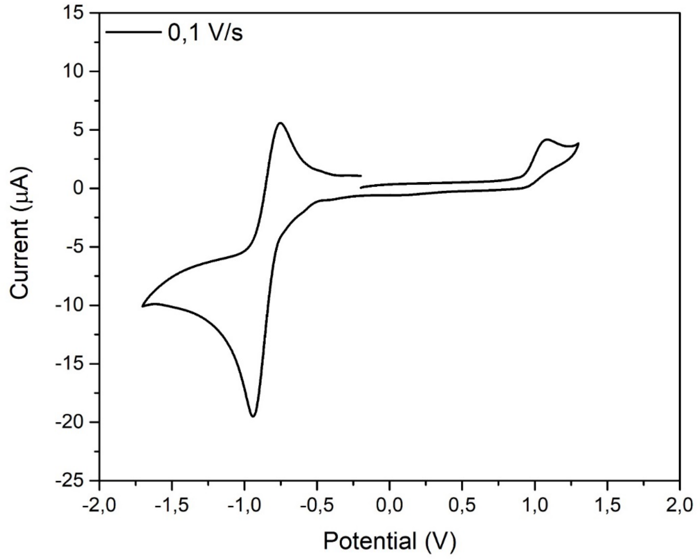

Figure S26. Cyclic voltammetry (CV) of **H_4PTCA** ligand in DMF using $TBAPF_6$ 0.1 M as electrolyte at 0.1 V/s scan rate. A platinum wire was used as the counter electrode and a silver wire as the pseudoreference electrode. Ferrocene was added as internal standard. All potentials are reported versus Ag/AgCl.

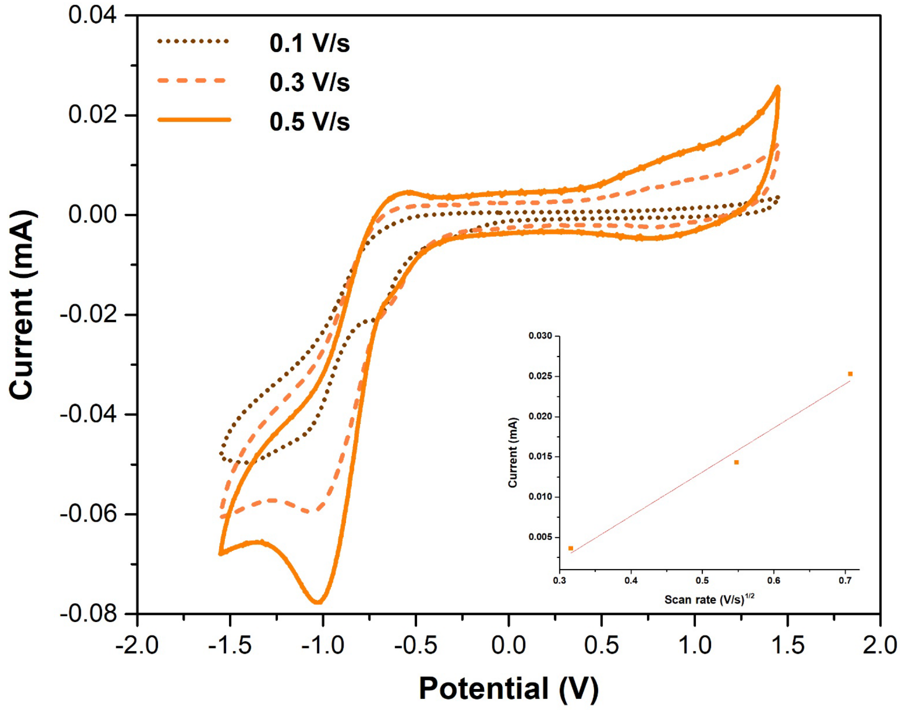

Figure S27. Solid-state cyclic voltammetry (CV) of **PTC-Co** CP in CH_3CN using $TBAPF_6$ 0.1 M as electrolyte at different scan rates. A platinum wire was used as the counter electrode and a silver wire as the pseudoreference electrode. Ferrocene was added as internal standard. All potentials are reported versus Ag/AgCl. The inset shows the linear relationship of cathodic peak current vs. scan rate.

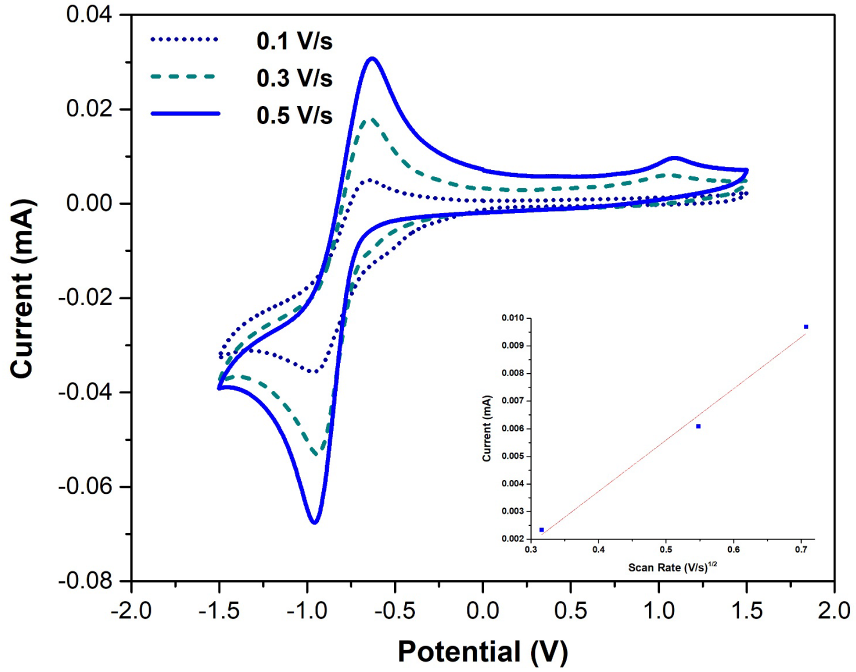

Figure S28. Solid-state cyclic voltammetry (CV) of **PTC-Ni** CP in CH_3CN using $TBAPF_6$ 0.1 M as electrolyte at different scan rates. A platinum wire was used as the counter electrode and a silver wire as the pseudoreference electrode. Ferrocene was added as internal standard. All potentials are reported versus Ag/AgCl. The inset shows the linear relationship of cathodic peak current vs. scan rate.

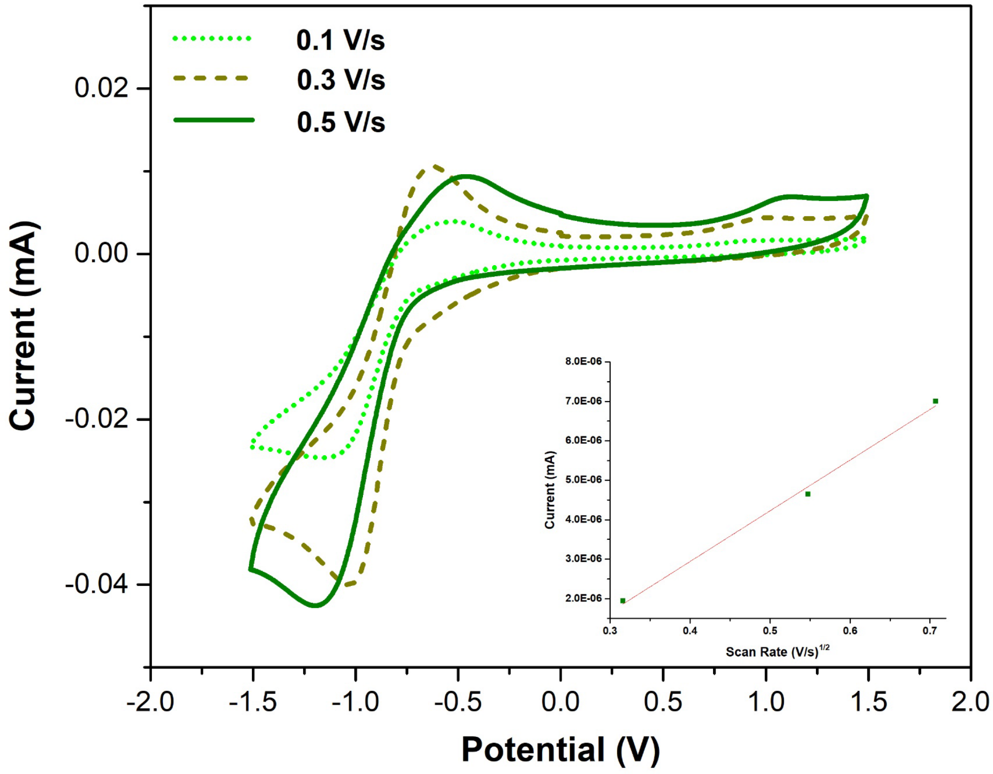

Figure S29. Solid-state cyclic voltammetry (CV) of **PTC-Zn** CP in CH_3CN using $TBAPF_6$ 0.1 M as electrolyte at different scan rates. A platinum wire was used as the counter electrode and a silver wire as the pseudoreference electrode. Ferrocene was added as internal standard. All potentials are reported versus Ag/AgCl. The inset shows the linear relationship of cathodic peak current vs. scan rate.

4. References

1. Digris, A. V.; Novikov, E. G.; Skakun, V. V.; Apanasovich, V. V.: Global Analysis of Time-Resolved Fluorescence Data. In *Fluorescence Spectroscopy and Microscopy: Methods and Protocols*; Engelborghs, Y., Visser, A. J. W. G., Eds.; Humana Press: Totowa, NJ, 2014; 257-277.
2. Coelho, A., *Topas Academic, Version 5.0, Coelho Software, Brisbane* **2013**.
3. Coelho, A., Indexing of powder diffraction patterns by iterative use of singular value decomposition. *J. Appl. Crystallogr.* **2003,** *36*, 86-95.
4. Wang, J.-M.; Yao, L.-Y.; Huang, W.; Yang, Y.; Liang, W.-B.; Yuan, R.; Xiao, D.-R. *ACS Appl. Mater. Interfaces* **2021**, *13*, 44079–44085.
5. Rietveld, H. M., A Profile Refinement Method for Nuclear and Magnetic Structures. *J. Appl. Crystallogr.* **1969,** *2*, 65-71.
6. Cheary, R. W.; Coelho, A., A FUNDAMENTAL PARAMETERS APPROACH TO X-RAY LINE-PROFILE FITTING. *Journal of Applied Crystallography* **1992,** *25*, 109-121.
7. Frisch, M. J.; Trucks, G. W.; Schlegel, H. B.; Scuseria, G. E.; Robb, M. A.; Cheeseman, J. R.; Scalmani, G.; V. Barone, G. A.; Petersson; Nakatsuji, H.; Li, X.; Caricato, M.; Marenich, A. V.; Bloino, J.; Janesko, B. G.; Gomperts, R.; Mennu, B.; Fox, D. J. Gaussian 16, Revision C. Wallingford CT 2016.
8. a) Perdew, J. P.; Ernzerhof, M.; Burke, K. Rationale for Mixing Exact Exchange with Density Functional Approximations. *J. Chem. Phys.* **1996**, *105* (22), 9982–9985; b) Adamo, C.; Barone, V. Toward Reliable Density Functional Methods without Adjustable Parameters: The PBE0 Model. *J. Chem. Phys.* **1999**, *110* (13), 6158–6170; c) Rassolov, V. A.; Ratner, M. A.; Pople, J. A.; Redfern, P. C.; Curtiss, L. A. 6-31G* Basis Set for Third-Row Atoms. *J. Comput. Chem.* **2001**, *22* (9), 976–984.
9. a) Blum, V.; Gehrke, R.; Hanke, F.; Havu, P.; Havu, V.; Ren, X.; Reuter, K.; Scheffler, M. Ab Initio Molecular Simulations with Numeric Atom-Centered Orbitals. *Comput. Phys. Commun.* **2009**, *180* (11), 2175–2196.b) R Havu, V.; Blum, V.; Havu, P.; Scheffler, M. Efficient Integration for All-Electron Electronic Structure Calculation Using Numeric Basis Functions. *J. Comput. Phys.* **2009**, *228* (22), 8367–8379; c) Ren, X.; Rinke, P.; Blum, V.; Wieferink, J.; Tkatchenko, A.; Sanfilippo, A.; Reuter, K.; Scheffler, M. Resolution-of-Identity Approach to Hartree–Fock, Hybrid Density Functionals, RPA, MP2 and GW with Numeric Atom-Centered Orbital Basis Functions. *New J. Phys.* **2012**, *14* (5), 053020.

10. Perdew, J. P.; Ruzsinszky, A.; Csonka, G. I.; Vydrov, O. A.; Scuseria, G. E.; Constantin, L. A.; Zhou, X.; Burke, K. Restoring the Density-Gradient Expansion for Exchange in Solids and Surfaces. *Phys. Rev. Lett.* **2008**, *100* (13), 136406.

11. Tkatchenko, A.; Scheffler, M. Accurate Molecular van Der Waals Interactions from Ground-State Electron Density and Free-Atom Reference Data. *Phys. Rev. Lett.* **2009**, *102* (7), 073005.

12. Krukau, A. V.; Vydrov, O. A.; Izmaylov, A. F.; Scuseria, G. E. Influence of the Exchange Screening Parameter on the Performance of Screened Hybrid Functionals. *J. Chem. Phys.* **2006**, *125* (22), 224106.

13. a) Jamorski, C.; Casida, M. E.; Salahub, D. R. Dynamic Polarizabilities and Excitation Spectra from a Molecular Implementation of Time-dependent Density-functional Response Theory: N 2 as a Case Study. *J. Chem. Phys.* **1996**, *104* (13), 5134–5147; b) Casida, M. E.; Jamorski, C.; Casida, K. C.; Salahub, D. R. Molecular Excitation Energies to High-Lying Bound States from Time-Dependent Density-Functional Response Theory: Characterization and Correction of the Time-Dependent Local Density Approximation Ionization Threshold. *J. Chem. Phys.* **1998**, *108* (11), 4439–4449; c) Petersilka, M.; Gossmann, U. J.; Gross, E. K. U. Excitation Energies from Time-Dependent Density-Functional Theory. *Phys. Rev. Lett.* **1996**, *76* (8), 1212–1215.

14. Momma, K.; Izumi, F. VESTA 3 for Three-Dimensional Visualization of Crystal, Volumetric and Morphology Data. *J. Appl. Crystallogr.* **2011**, *44* (6), 1272–1276.

15. Chemcraft - graphical software for visualization of quantum chemistry computations. https://www.chemcraftprog.com
